# An Additional Baurusuchid from the Cretaceous of Brazil with Evidence of Interspecific Predation among Crocodyliformes

**DOI:** 10.1371/journal.pone.0097138

**Published:** 2014-05-08

**Authors:** Pedro L. Godoy, Felipe C. Montefeltro, Mark A. Norell, Max C. Langer

**Affiliations:** 1 Departamento de Biologia, Faculdade de Filosofia, Ciências e Letras de Ribeirão Preto – Universidade de São Paulo, Ribeirão Preto, São Paulo, Brazil; 2 Division of Paleontology, American Museum of Natural History, New York, New York, United States of America; 3 Departamento de Zoologia, Universidade Estadual Paulista (UNESP), Rio Claro, São Paulo, Brazil; Team ‘Evo-Devo of Vertebrate Dentition’, France

## Abstract

A new Baurusuchidae (Crocodyliformes, Mesoeucrocodylia), *Aplestosuchus sordidus*, is described based on a nearly complete skeleton collected in deposits of the Adamantina Formation (Bauru Group, Late Cretaceous) of Brazil. The nesting of the new taxon within Baurusuchidae can be ensured based on several exclusive skull features of this clade, such as the quadrate depression, medial approximation of the prefrontals, rostral extension of palatines (not reaching the level of the rostral margin of suborbital fenestrae), cylindrical dorsal portion of palatine bar, ridge on the ectopterygoid-jugal articulation, and supraoccipital with restricted thin transversal exposure in the caudalmost part of the skull roof. A newly proposed phylogeny of Baurusuchidae encompasses *A. sordidus* and recently described forms, suggesting its sixter-taxon relationship to *Baurusuchus albertoi*, within Baurusuchinae. Additionally, the remains of a sphagesaurid crocodyliform were preserved in the abdominal cavity of the new baurusuchid. Direct fossil evidence of behavioral interaction among fossil crocodyliforms is rare and mostly restricted to bite marks resulting from predation, as well as possible conspecific male-to-male aggression. This is the first time that a direct and unmistaken evidence of predation between different taxa of this group is recorded as fossils. This discovery confirms that baurusuchids were top predators of their time, with sphagesaurids occupying a lower trophic position, possibly with a more generalist diet.

## Introduction

Crocodyliformes, the group that includes modern crocodiles and their extinct relatives, were much more diverse in the past than today. [1]. Several different environments were occupied by a variety of taxa, including fully marine, robust and gracile terrestrial forms, with distinct inferred niche partitioning [2], [3]. The Late Cretaceous Adamantina Formation, in south-central Brazil, has yielded the most diverse known crocodyliform fauna, with twenty described species. This includes not only members of the trematochampsid-peirosaurid clade [4], [5], with their semiaquatic body-plan, but also an array of more terrestrial forms, such as large-sized baurusuchid predators [6], and the bizarre notosuchids and sphagesaurids [7], [8]. The latter two groups depart from the typical carnivorous habits of crocodyliforms, with adaptations that suggest omnivorous or even herbivorous diets [3].

**Figure 1:**
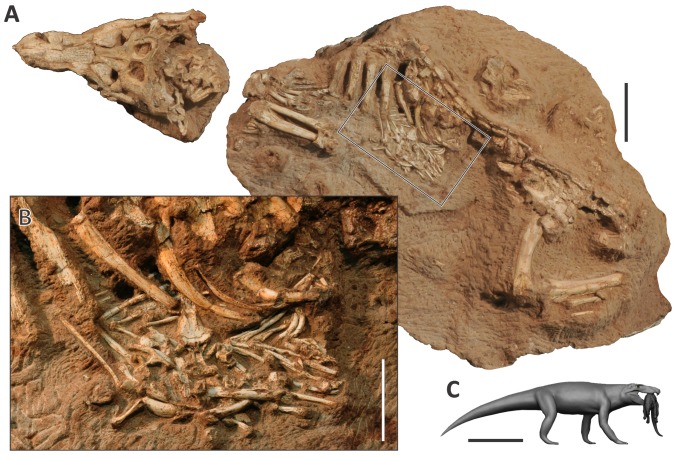
Fossil crocodyliformes showing predator-prey interaction. A, *Aplestosuchus sordidus* skeleton (LPRP/USP 0229a). Scale bar, 10 cm. B, Area highlighted in “a” with details of the abdominal content, including sphagesaurid remains (LPRP/USP 0229b). Scale bar, 5 cm. C, Reconstructed predator and prey. Reconstruction by Rodolfo Nogueira. Scale bar, 50 cm.

Despite the richness of the group in Mesozoic-Cenozoic deposits, direct fossil evidence of behavioral interaction is rare for extinct crocodyliforms. The fossil record is restricted to bite marks showing that carnivorous forms preyed on other crocodyliforms [9], and other tetrapods, like dinosaurs and turtles [10], [11], or suggesting aggression between individuals of the same species [12]–[15]. Here we report the first record of unmistaken abdominal contents, or cololites, preserved for a fossil crocodyliform. It corresponds to a new baurusuchid species, with the remains of another crocodyliform, a sphagesaurid, in its abdominal cavity (Figure 1). These fossils unequivocally indicate, for the first time, direct interaction (predation) between two different species of Crocodyliformes.

## Materials and Methods

### Tree search and support measurements

The phylogenetic dataset was analyzed using equally weighted parsimony in TNT [16], based on an implicit enumeration search. Bootstrap (GC frequencies >50%) and Bremer support values [17] were calculated for the data in hand using TNT v.1.1; the first employing the TNT script available, and the latter generated from the resampling routine command with 1,000 bootstrap pseudoreplicates.

### Nomenclatural Acts

The electronic edition of this article conforms to the requirements of the amended International Code of Zoological Nomenclature, and hence the new names contained herein are available under that Code from the electronic edition of this article. This published work and the nomenclatural acts it contains have been registered in ZooBank, the online registration system for the ICZN. The ZooBank LSIDs (Life Science Identifiers) can be resolved and the associated information viewed through any standard web browser by appending the LSID to the prefix “http://zoobank.org/”. The LSID for this publication is: urn:lsid:zoobank.org:pub:B67AF736-90B3-4A5C-ACE5-131340B63C3A. The electronic edition of this work was published in a journal with an ISSN, and has been archived and is available from the following digital repositories: PubMed Central, LOCKSS.

### Fieldwork permit and repository information

All necessary permits were obtained for the described study, which complied with all relevant regulations. The field work and fossil collection was previously communicated to the Departamento Nacional de Produção Mineral — DNPM, as requested in the ordinance n° 4.146 from March 4th, 1942. The specimens described in this work are deposited in the permanent collection of Laboratório de Paleontologia, Universidade de São Paulo (Ribeirão Preto, Brazil).

### Systematic paleontology

**Crocodyliformes** Benton & Clark 1988 [18]

**Mesoeucrocodylia** Whetstone & Whybrown 1983 [19]

**Baurusuchidae** Price, 1945 [20]

**Aplestosuchus gen. nov.**

urn:lsid:zoobank.org:act:E12B92AE-CD8B-4351-9F2E-36BEE125E38D

**Etymology.** From *Aplestos* (Gr.), insatiate, gluttonous, and *souchos* (Gr.), after a creature of Egyptian zoomorphism.

**Type species.** *Aplestosuchus sordidus* gen. et. sp. nov.

urn:lsid:zoobank.org:act:3852F31C-E337-4F55-9338-F0510032A9AE

**Etymology.** *sordidus* (L.), filthy; in reference to the manifest greedy behavior of the animal.

**Holotype.** LPRP/USP (Laboratório de Paleontologia, Universidade de São Paulo) 0229a is an articulated, nearly complete skeleton (Figure 1A).

**Figure 2:**
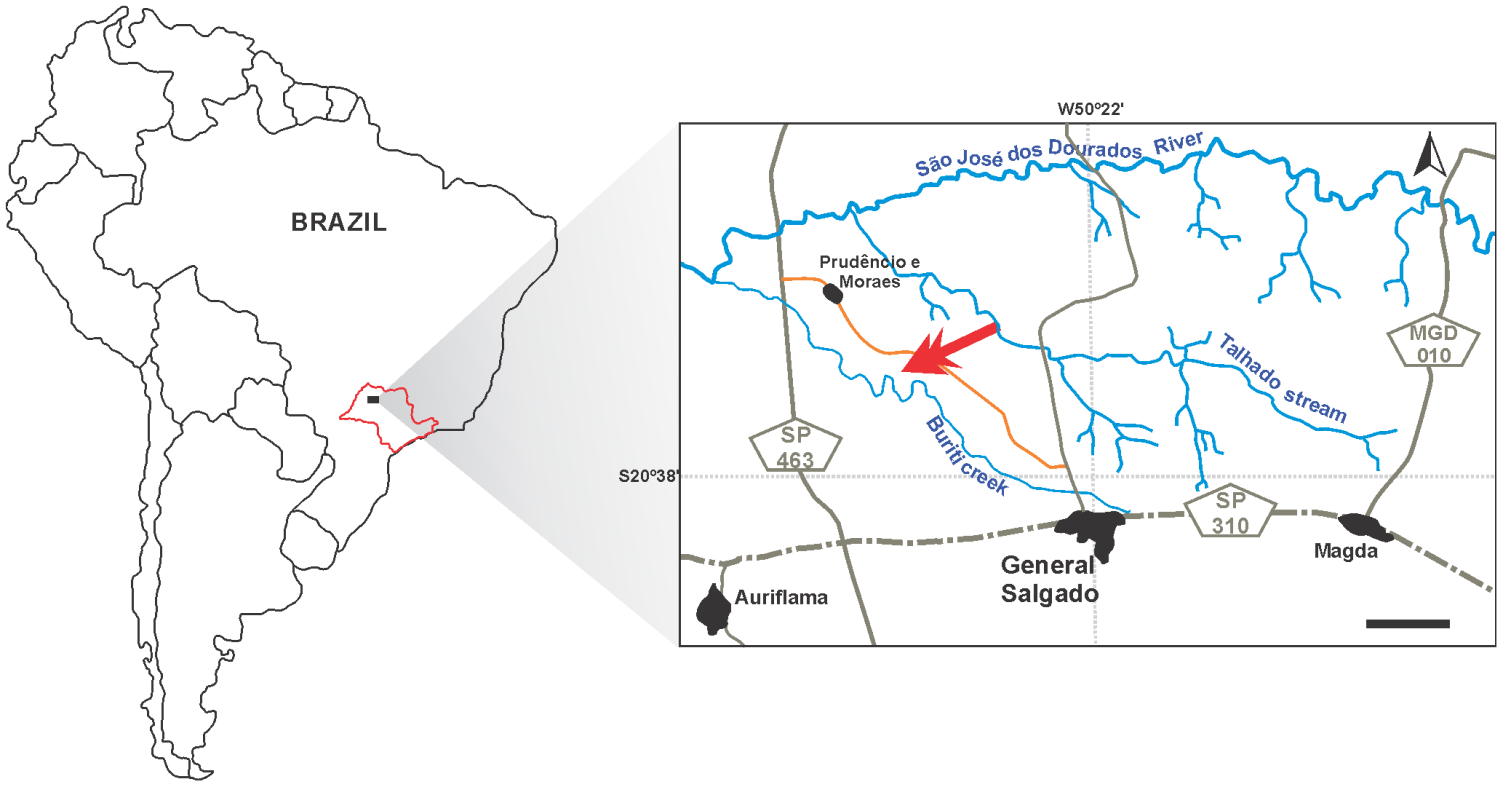
Map showing the location where *Aplestosuchus sordidus* was collected within South America and São Paulo State (Brazil). Watercourses in blue, paved roads in grey, nonpaved roads in orange, cities and towns in black. The red arrow indicates the outcrop area. Scale bar equals 3

**Type locality.** Buruti creek area, General Salgado municipality, São Paulo, Brazil (20°34′ 0″ S; 50°27′ 55″ W) (Figure 2). This is the same area yielded the type-specimens of four other crocodyliforms: *Baurusuchus albertoi*, *B. salgadoensis*, *Armadillosuchus arrudai*, and *Gondwanasuchus scabrosus* [21]–[24].

**Age and horizon.** Adamantina Formation, Bauru Group (Late Cretaceous of the Paraná Basin) [25].

**Diagnosis.** Distinguished from all other known Crocodyliformes by the following unique set of traits (autapomorphies marked with asterisks): nasal with dorsal midline crest*, frontal longitudinal ridge reaching the midline contact between prefrontals*, crest-shaped medial supratemporal rim, ridge along the ectopterygoid-jugal suture notched at its caudal portion, lateral depression on quadrate, palatine bar with crested ventral surface* and cylindrical dorsal portion, choanal septum with ridged ventral surface, single parachoanal fossa rostrolateral to the parachoanal fenestrae at the base of the pterygoid wing*, outer sculpture of the mandible restricted to the dentary, no peg at the occipital surface of the mandibular symphysis, ridged border of the angular not covering the rostral edge of the mandibular fenestra, row of foramina between the mandibular fenestra and the ectopterygoid-jugal suture.

**Abdominal contents.** LPRP/USP 0229b, isolated teeth and skull bones of a Sphagesauridae (Figure 1B).

## Description

### Aplestosuchus sordidus

More than one meter long from the tip of the skull to the base of the tail, *Aplestosuchus sordidus* was preserved lying on its side (Figure 1A), a stereotypical death pose also found in other baurusuchids of the site [21], [26]. It suffered some post-mortem disarticulation, as most of the tail and the distal parts of the hindlimbs were lost (Figure 1A). LPRP/USP 0229a is preserved in two different blocks. The skull articulates with the first five cervical vertebrae, three of them with cervical ribs. The neural arches of both atlas and proatlas are visible. The axis is turned to the left, so its neural spine is horizontally disposed. This rotation is also seen in the other three cervical vertebrae (III, IV and V). The remaining elements of the postcranium are isolated in a larger block, in which almost the entire trunk vertebral series is exposed, with a single line of paramedial osteoderms and a nearly intact rib case. The articulated right radius and ulna are exposed, as well as elements of both manus. Part of the left pelvic girdle is exposed in dorsal view, as are the left femur, tibia, and fibula.

**Figure 3:**
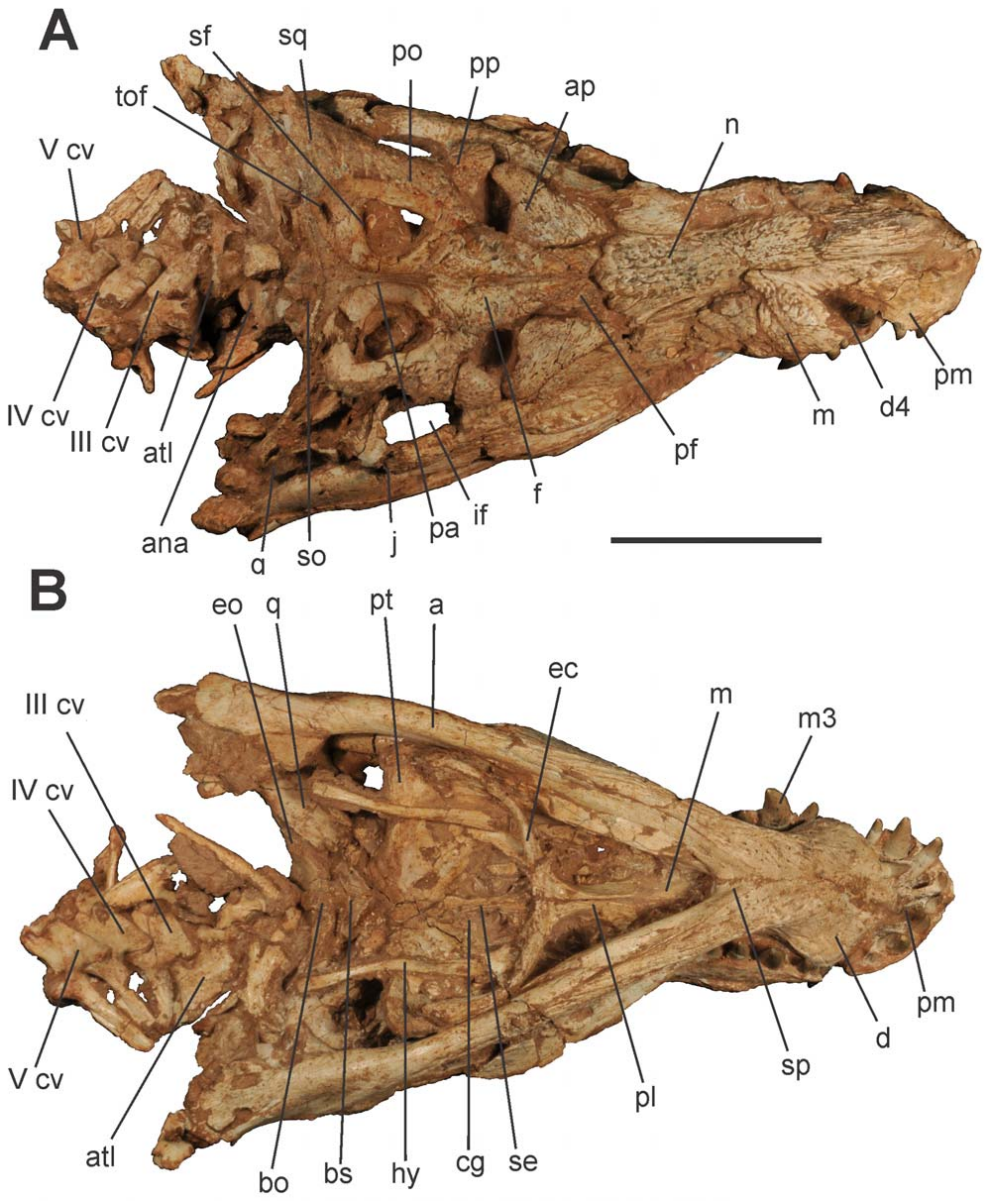
Skull of *Aplestosuchus sordidus* LPRP/USP 0229a in A, dorsal view; and B, ventral. Abbreviations: a, angular; ap, anterior palpebral; ana, atlantal neural arch; atl, atlas; bo, basioccipital; bs, basisphenoid; cg, choanal groove; d, dentary; d4, dentary tooth 4; ec, ectopterygoid; eo, exoccipital; f, frontal; hy, hyoid aparatus; if, infratemporal fenestra; j, jugal; m, maxilla; m3, maxillary tooth 3; n, nasal; pa, parietal; pf, prefrontal; pl, palatine; pm, premaxilla; po, postorbital; pp, posterior palpebral; pt, pterygoid; q, quadrate; se, choanal septum; sf, supratemporal fenestra; so, supraoccipital; sq, squamosal; sp, splenial; tof, temporo-orbital foramen; III–V cv, cervical vertebrae III–V. Scale bar equals 10 cm.

**Figure 4:**
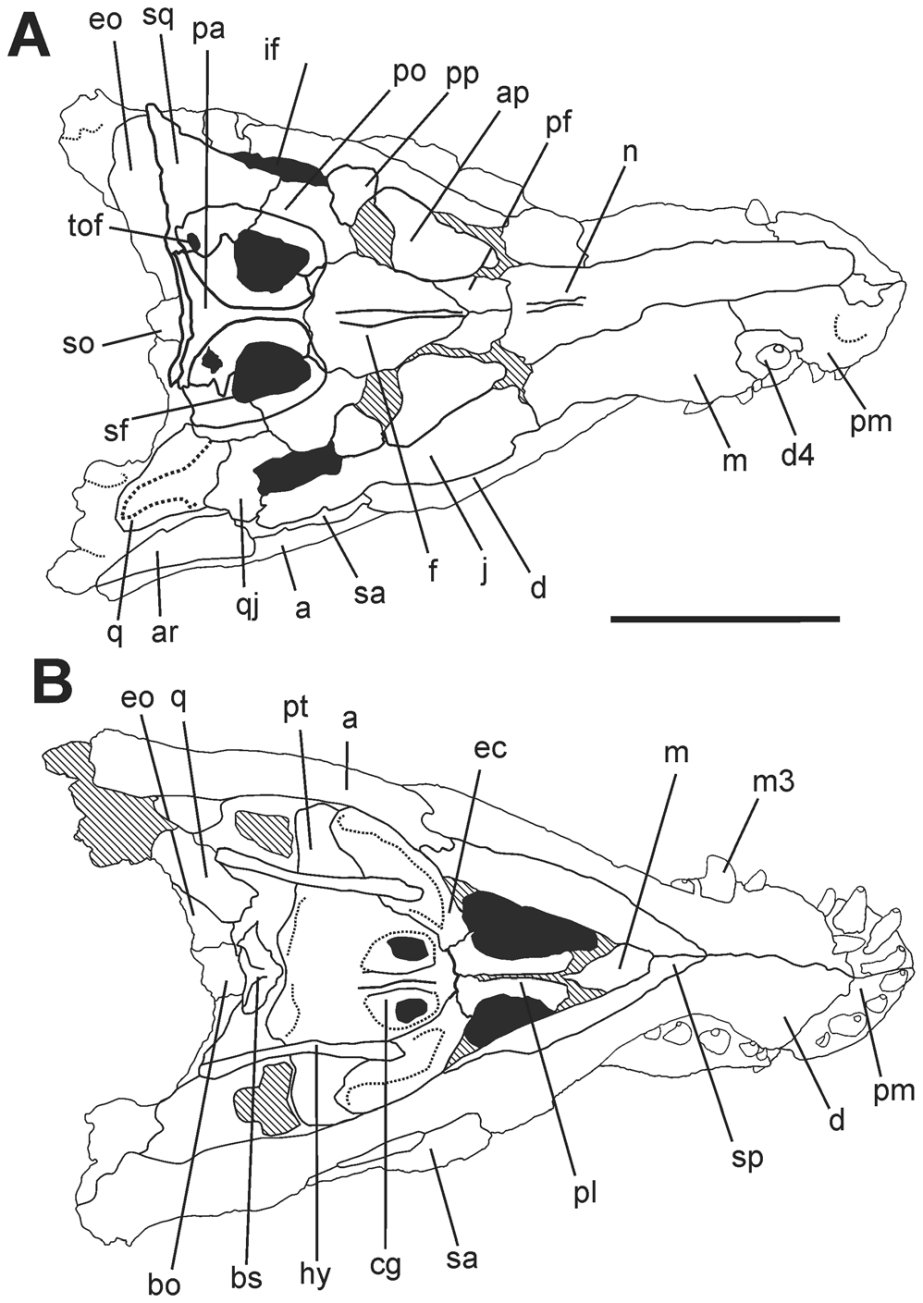
Drawing of the skull of *Aplestosuchus sordidus* LPRP/USP 0229a in A, dorsal view; and B, ventral. Abbreviations: a, angular; ap, anterior palpebral; ar, articular; bo, basioccipital; bs, basisphenoid; cg, choanal groove; d, dentary; d4, dentary tooth 4; ec, ectopterygoid; eo, exoccipital; f, frontal; hy, hyoid aparatus; if, infratemporal fenestra; j, jugal; m, maxilla; m3, maxillary tooth 3; n, nasal; pa, parietal; pf, prefrontal; pl, palatine; pm, premaxilla; po, postorbital; pp, posterior palpebral; pt, pterygoid; q, quadrate; qj, quadratojugal; sa, surangular; sf, supratemporal fenestra; so, supraoccipital; sq, squamosal; sp, splenial; tof, temporo-orbital foramen. Scale bar equals 10 cm.

**Figure 5:**
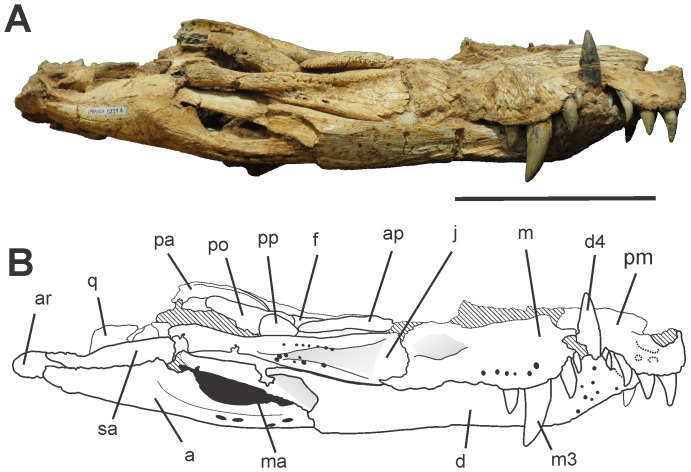
Skull of *Aplestosuchus sordidus* LPRP/USP 0229a in lateral (right) view. A, photograph. B, interpretative drawing. Abbreviations: a, angular; ap, anterior palpebral; ar, articular; d, dentary; d4, dentary tooth 4; f, frontal; j, jugal; m, maxilla; ma, mandibular fenestra; m3, maxillary tooth 3; pa, parietal; pm, premaxilla; po, postorbital; pp, posterior palpebral; q, quadrate; sa, surangular. Scale bar equals 10 cm.

The skull is nearly complete with articulated lower jaws (Figures 3, 4 and 5). It is dorsoventraly compressed, exposing the otic recesses and the infratemporal fenestrae in dorsal view, and hiding the orbits, lacrimals, postorbital bars, and most of the quadratojugals (Figures 3A and 4A). Irregular pits and ridges cover the external surface of most dermal bones.

The external supratemporal fenestra (*sensu* [6]) occupies a great portion of the skull roof. Its margins are composed by the frontal, postorbital, squamosal, and parietal bones, the last of which demarcates restricted caudal and medial margins. The rostral margins are not hypertrophied, a condition shared with all Baurusuchinae, one of the two main lineages within Baurusuchidae [6]. The infratemporal fenestra is composed by the jugal, quadratojugal, and likely the postorbital, although the dorsoventral compression has hidden the contribution of the latter bone. The antorbital fenestrae and fossa are completely obliterated.

As a result of the dorsoventral compression of the skull, the septate external nares are only partially preserved. The septum is formed by the premaxillae ventrally, and the nasal dorsally, but the exact contribution of each bone is not possible to determine. Together, these bones most likely separated the nares completely. Each premaxilla bears a large perinarial depression lateral to each nostril. Just ventral to the depression there are two foramina piercing the premaxilla above the third premaxillary tooth, as in *Pissarrachampsa sera* [6] and *Baurusuchus salgadoensis* [22], but distinct from *Wargosuchus australis* [27], which bears three foramina on the surface of the perinarial depression.

The premaxilla-maxilla suture is similar to the general condition seen in other baurusuchids. It is partially hidden in the premaxilla-maxilla notch that receives the fourth dentary tooth. At the dorsalmost portion of this notch, the suture extends dorsally, with a slight caudal deflection next to the triple contact between premaxilla, maxilla, and nasal. Medially, the premaxilla-nasal suture starts at the caudolateral margin of the nostril and extends caudolaterally up to the notch for the fourth dentary tooth.

The maxilla forms most of the lateral surface of the rostrum and probably had a nearly vertical orientation in its original position (Figure 5). In addition, the maxillae would not significantly contribute to the dorsal surface of rostrum. The combination of these features categorizes *Aplestosuchus sordidus* as an oreinirostral taxon. The maxilla contacts the nasal medially, the jugal caudally, and supposedly the lacrimal at the level of palpebral supporting structures, since this latter bone is not exposed due to the compression of the skull. Even though the orbits were collapsed, the complete caudal edge of the maxilla is visible, and it does not take part on the orbital margin. The same condition is seen in *Baurusuchus pachecoi*, *B. salgadoensis*, *Stratiotosuchus maxhechti*, and *Pissarrachampsa sera* [6], [20], [22], [28]. Although mostly hidden by the occluded mandible, the palatal shelves of the maxillae are visible in ventral view, forming the rostral portion of the secondary bony palate. The palatal maxilla-palatine suture is transversely oriented, caudal to the rostral edge of suborbital fenestra, and spanning from the midline to the medial borders of that aperture (Figures 3B and 4B).

The nasal forms most of the dorsal surface of the snout, but has virtually no contribution to its lateral surface. There is no external vestige of a midline suture between the nasals, a condition seen in all baurusuchids, except *Gondwanasuchus scabrosus* [24]. The caudalmost portion of each bone is rounded and lacks the longitudinal depression next to the medial contact of the prefrontals. The same condition is seen in *Baurusuchus salgadoensis* and *Stratiotosuchus maxhechti* [22], [28], but not in pissarrachampsines, which bear the depression [6]. Furthermore, the dorsal surface of the nasal has a midline crest, an unique trait of *Aplestosuchus sordidus*. This crest starts at the mid portion of the nasal and extends caudally to meet the frontal median ridge at the level of the midline contact between the prefrontals.

The anterior palpebral is hook-shaped, as in *Baurusuchus salgadoensis*, *Pissarrachampsa sera*, and *Gondwanasuchus scabrosus* [6], [22], [24]. Its lateral margin extends caudally and contacts a short rostrolateral process of the posterior palpebral. The dorsal surfaces of the anterior palpebral and prefrontal form a continuous plane, differently from the condition in *P. sera* and *Wargosuchus australis* [6], [27]. Both palpebral bones conceal a well-developed rounded foramen on the roof of the orbit.

The prefrontal has a large dorsal exposure, but its participation on the support structures for anterior palpebral is not accessible. In dorsal view, the medial approximation of the prefrontals is formed by a short medial contact. The medial approximation of prefrontals is a baurusuchid synapomorphy, present in three different states within the group. The condition of *Aplestosuchus sordidus* is intermediate between pissarrachampsines and baurusuchines. The approximation is not limited to a single medial point, as in *Pissarrachampsa sera*, *Wargosuchus australis*, and *Gondwanasuchus scabrosus* [6], [24], [27], but is less developed than in *Baurusuchus salgadoensis* and *Stratiotosuchus maxhechti*, in which most of the medial edges of the prefrontals are tightly sutured [22], [28].

The frontal extends rostrally between the prefrontal pair. The orbital margin is slightly convex, forming the lateral margin of the foramen shaped by the palpebrals. From the caudal orbital edge, the frontal extends caudomedially, entering the supratemporal fossa, but not reaching the edge of the internal supratemporal fenestrae. Caudally, the frontal contacts the parietal in a roughly transverse suture at the dorsal roof, before entering the supratemporal fossa laterally. Its outer surface is mostly unornamented, but bears a well-developed midline crest also seen in *Stratiotosuchus maxhechti*, *Pissarrachampsa sera*, and *Baurusuchus salgadoensis,* but less developed in the latter [6], [28], [22]. In *A. sordidus* this crest is unique because extends from the level of orbital caudal edge to the level of the prefrontal medial contact. The general surface of frontal is flat, similarly to that of *P. sera* and *Campinasuchus dinizi* [6], [29], but differently from the strongly transversally bowed frontal surface of *B. salgadoensis* and *S. maxhechti* [22], [28]. In *Aplestosuchus sordidus* the frontal width between the orbits only slightly surpasses the nasal width, as in *B. salgadoensis* and *S. maxhechti* [22], [28]. On the other side, in *P. sera* and *Campinasuchus dinizi*, the frontal width is twice the nasals width [6], [29].

The jugal is a long and transversely narrow bone forming most of the cheek region of *Aplestosuchus sordidus*. Its infraorbital process expands dorsoventrally, but the extent of this expansion is obscured by the deformation of the skull. As in other baurusuchids, the jugal infraorbital ramus has a lateral hypertrophied infraorbital ridge (*sensu* [6]), extending rostrally up to the level of the rostral margin of the orbit and caudally along the entire infratemporal ramus. Also as in other baurusuchids, a fan-shaped depression with a sculpted surface is seen ventrally to the infratemporal ridge. At the ventral portion of this depression, a series of six well developed neurovascular foramina is noted, a condition shared only with *Baurusuchus albertoi* and *Gondwanasuchus scabrosus* [21], [24]. The infratemporal ridge converges ventrocaudally to the ridged jugal-ectopterygoid suture, at the level of the junction of the three jugal processes. In this area, there is a distinguishable jugal notch (*sensu* [6]), a feature shared with *B. albertoi*, *Stratiotosuchus maxhechti*, and *Pissarrachampsa sera* [6], [21], [28].

The postorbital forms the rostrolateral corner of the skull roof and the rostral portion of the bar that separates the orbit from internal and external supratemporal fenestrae. Along with the squamosal, it forms the lateral shelf overhanging the auditory meatus. The postorbital- squamosal contact forms a straight line in lateral view, as seen in *Stratiotosuchus maxhechti* [28]. The rostrolateral corner of postorbital supports the posterior palpebral, but its articulation facet is not exposed. The postorbital presumably possess a well-developed descending process that contributes to the postorbital bar, but this is obscured due to the compression of the skull. In the same way, the contribution of the postorbital within the auditory meatus and its contact to the quadratojugal is not accessible.

The squamosal forms the caudolateral corner of the skull roof and deflects ventrally, as an oblique plate that extends parallel to the dorsal edge of the paroccipital process, enclosing the otic recess caudally. As in other baurusuchids, this bone has a distinctly pebbled surface at the border of the external supratemporal fenestra. In lateral aspect, its caudoventral edge curves slightly over the otic recess. The caudal portion of that surface bears a shallow longitudinal groove, which also extends ventrally along the deflected process of the squamosal, enclosing the otic recess caudally. This groove is probably related to the attachment of the hinge ligament of the auricular plate associated with the upper earflap [30], [31], [32], and the same condition is shared by other baurusuchids. Rostrodorsally, the squamosal is sutured to the postorbital about the mid-length of the external supratemporal fenestra.

The parietal is restricted to the medial margin of the external and internal supratemporal fenestrae. This forms a sagittal crest without the hypertrophied rims seen in *Baurusuchus salgadoensis* and *Stratiotosuchus maxhechti* [22], [28]. Within each supratemporal fossa, the parietal contacts the postorbital ventrally, excluding the frontal from the internal supratemporal fenestra, as in pissarrachampsines and *B. salgadoensis* [6], [22]. Caudolaterally, the parietal contacts the squamosal at the caudal margin of the internal and external supratemporal fenestrae. A large temporo-orbital foramen is located on the suture between these two bones. The caudal margin of the parietal is firmly attached to the supraoccipital, excluding the former bone from the caudal margin of the skull.

The pterygoid is only accessible in palatal view, and its possible contacts with the prefrontal pillar and parasphenoid are hidden (Figures 3B and 4B). The palatine palatal shelves are sutured into a single element that forms the caudal half of the secondary bony palate. Dorsally, the palatine is cylindrical with a nearly entirely unsculptured surface. The ventral surface of the palatine bar forms a sharp crest along most of its length, differing from all other higher baurusuchids [6], in which that surface is flat thorough. Accordingly, the rugose midline contact of the palatines and the flanking rows of foramina, typical of higher baurusuchids [6], are only seen caudally, near the rostral border of the choana. Its crest-shaped ventral surface gives the palatine a more slender appearance than in other baurusuchids. In the same way, the caudolateral processes of the bone is more rostrocaudally restricted than in other members of the group. These processes firmly attach to the rostromedial process of the ectopterygoid and bound the rostral apex of the wide choanal aperture. The palatine-pterygoid contact is not visible in palatal view, but considering the configuration of the bones, this contact is probably rostral to the choanal border.

The pterygoids form the greatest part of a large choanal depression, which is also bound rostrally by the palatines and rostrolaterally by the ectopterygoids. As in other baurusuchids, the palatine-ectopterygoid margin of the choana is ventrally located in relation to the remaining parts, so that the depression opens caudoventrally. The choanal depression bears a divided choanal groove and a series of parachoanal structures. The subcolumnar choanal septum is formed entirely by the pterygoids and has a marked ridged ventral surface. A subcircular parachoanal fenestra is present in the rostrolateral portion of each pterygoid, lateral to the choanal septum. These fenestrae are laterally and caudally enclosed by the expanded pterygoid choanal roof, rostrally by the palatine and rostrolaterally by the ectopterygoid. Laterally to the parachoanal fenestra, at the base of the pterygoid wing, a single broad parachoanal fossa excavates the pterygoid. This configuration of parachoanal structures is unique among crocodyliforms with parachoanal fenestrae and fossae (Sphagesauridae plus Baurusuchidae) [6], [7], [33]. The pterygoid wing of *Aplestosuchus sordidus* is overlapped by the ectopterygoid ventrally. Yet, the latter is restricted to the lateral portion of the wing and does not reach its caudoventral tip. Caudally to the choanal opening, the pterygoid does not form a vertical wall, but the pterygoid-basisphenoid tuber is firmly attached to the rostral portion of the basisphenoid. The pterygoid-quadrate contact is obliterated by cracks and fragments, but it seems to be at the level of the basioccipital lateral extension.

The ectopterygoid forms the caudolateral portion of choanal depression. This bone is restricted to a limited ridge at the lateral surface of the pterygoid wing. The ectopterygoid body is oriented rostromedially and has an elliptical cross-section. In ventral view, the bone meets the caudolateral process of the palatine perpendicular to the rostrocaudal axis of the skull in ventral view.

Only small portions of the quadrates are exposed. This bone forms the jaw joint and is sutured to the lateral portion of the braincase. Its original position cannot be determined due to the flattening of the skull, but it certainly had a more vertical orientation. The outer surface of the quadrate has a depression near the articular condyle, as seen in all baurusuchids with this area preserved [6]. The right otic aperture is partially exposed and it is as large as in other baurusuchids. The quadrate otic region bears additional fenestration, but the bad preservation precludes a detailed description of these apertures. The rostral contact of the quadrate with the braincase wall is severed damaged.

The neurocranium is heavily damaged due to the fragmentation of the occipital region. The supraoccipital can be seen on the dorsal surface of the skull as a single thin slip, articulated to the caudal margin of the parietal. It tapers laterally until its inflexion to the occipital surface, where its contact to the surrounding bones cannot be accessed. The occipital surface of the supraoccipital is, however, partially seen. This formed a roughly vertical wall in its original position and its dorsal half bears a low median ridge. The exoccipital and opisthotic are fused into the otoccipital, which forms most of the lateral portion of the occipital plane. Its participation in the foramen magnum and occipital condyle cannot be accessed, neither the paths of cranial nerves and vessels at the occipital plane. In ventral view, the caudal surface of the basioccipital is slightly inclined and faces caudoventrally. A slight median crest extends in this surface, from the presumable condylar neck region to the suture with the basisphenoid, but the openings of the Eustachian tubes are not preserved. The basisphenoid is largely exposed in ventral aspect, bound by the pterygoids rostrally and basioccipital caudally. The articulation of its lateral projection to pterygoid and quadrate are not preserved. As in other baurusuchids, the basisphenoid body is set at a dorsal position and separated from the ventral surface of the surrounding bones by a well-developed step [6]. The ventral surface of the basisphenoid has two well-defined crests converging rostrally.

Both hyoids of *Aplestosuchus sordidius* are preserved in an assumed approximate life position: semi-paralleled orientation and lateral to the choanal groove. These elements are slim, slightly curved outwards and expanded in the extremities. In ventral view, they span from the level of the parachoanal fossae to that of the caudal limit of the braincase.

Both madibular rami, with dentary, splenial, surangular, angular and articular, are preserved. The dentary forms most of the lateral surface of the mandible (Figure 5). It has several pits and grooves, mainly in the symphyseal region. In lateral view, the ventral margin of the dentary extends parallel to the long axis of the lower jaw up to the level of the third dentary tooth. Rostral to this point, the dentary turns up in a rostrodorsal extension that forms an angle of approximately 45° to the mandible long axis. The mandibular symphysis is relatively long and extends until the level of the fifth maxillary tooth. There is a marked depression on its ventral surface, near the dentary-splenial contact. This depression is homologous to the dorsalmost and more restricted depression of the symphyseal depressions in *Pissarrachampsa sera* [6]. It extends caudally, as a thin groove, along the contact between dentary and splenial, and gets smoother until it disappears at the level of the last maxillary tooth. Although deformed by the dorsoventral compression, the foramen intermandibularis oralis was probably large and slot-like. Also due to this compression, most of the caudal portion of the medial surface of the mandible is concealed. In lateral view, a large external mandibular fenestra is enclosed by the caudal portion by dentary, the angular and surangular. Below the fenestra, there is a row of large nutritional foramina, as in *Baurusuchus albertoi* [21]. In ventral view, the caudalmost portion of the angular expands lateromedially. The small visible portion of the articular bears a well-developed retroarticular process projecting ventrocaudally, as in *B. salgadoensis* and *B. albertoi* [21], [22].

**Figure 6:**
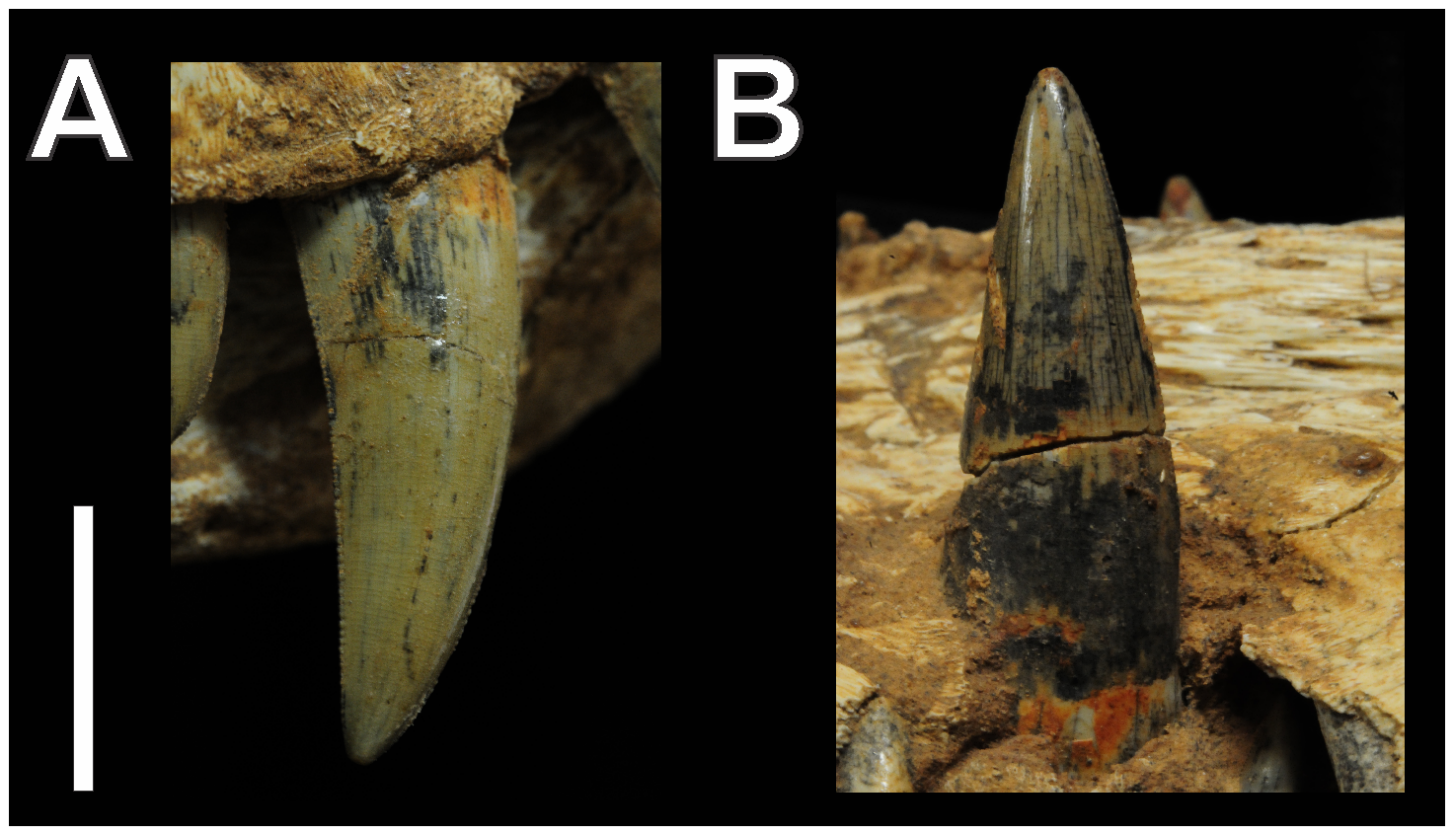
The largest teeth of *Aplestosuchus sordidus*. A, Third maxillary (right) tooth; B, Fourth dentary (right) tooth. Scale bar equals 2 cm.

*Aplestosuchus sordidus* has a reduced number of teeth, four in each premaxilla and five in each maxilla. The total number of dentary teeth is not preserved, but comparisons to other baurusuchids suggest no more than nine. The premaxillary and rostral dentary teeth have round cross-sections and are slightly caudally curved, lacking well-defined carinae. The maxillary teeth and the non-symphyseal accessible dentary teeth are ziphodont (*sensu* [34]): serrated, labiolingually compressed, and caudally curved. The first, second and fourth premaxillary teeth are of similar size, whereas the third is hypertrophied (Figures 3B and 4B). Concerning the maxillary teeth, the first is the smallest, the third is the largest, and the remaining have about the same size (Figure 6A). The first dentary tooth is procumbent and fits in to a slit in the premaxillary palatinal shelves, between the first and second premaxillary alveolus. The fourth dentary tooth is the largest of the series, and fits in the premaxillary-maxillary notch (Figure 6B).

### Abdominal contents

**Figure 7:**
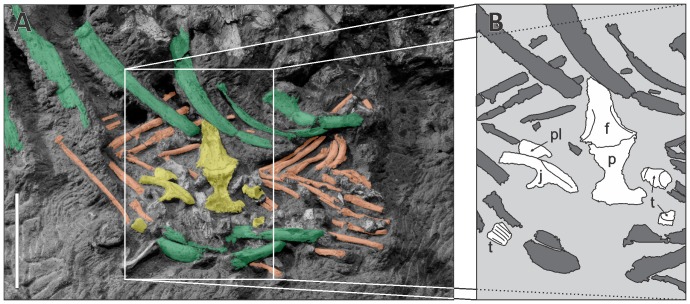
Abdominal contents (LPRP/USP 0229b). A, Photograph depicting ribs (green) and gastralia (orange) of *Aplestosuchus sordidus*, and sphagesaurid remains (yellow). Scale bar, 5 cm. B, Detail of the area highlighted in “A”, with sphagesaurid remains identified in white. Abbreviations: f, frontal; j, jugal; p, parietal; pl, palpebral; t, tooth.

**Figure 8:**
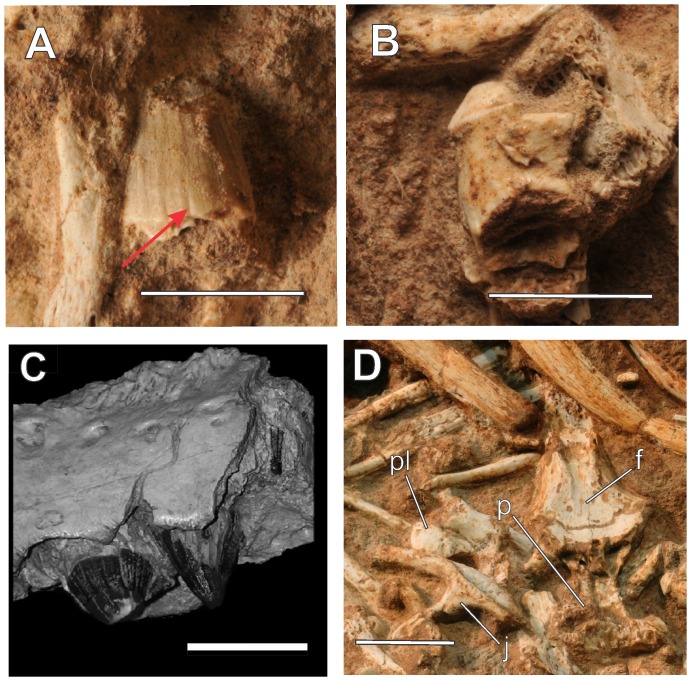
Details of the preserved remains of LPRP/USP 0229b. A, B, Teeth of in lateral view. The red arrow in “A” indicates one of the basiapical keels, typical for Sphagesauridae. C, Detail of the teeth of another Sphagesauridae, *Caryonosuchus pricei*, modified from [35]. D, Cranial bones preserved. Abbreviations: f, frontal; j, jugal; p, parietal; pl, palpebral. Scale bars, 10 mm.

During preparation, a cluster of small bones was found ventral to the preserved ribs of *Aplestosuchus sordidus* (LPRP/USP 0229a) (Figure 7). Some of these elements are articulated gastralia of that same individual. Yet, some extrinsic elements (LPRP/USP 0229b) are also present, including three isolated teeth, as well as frontal, parietal, palpebral, and jugal bones (Figure 8). The teeth are typical of sphagesaurid crocodyliforms, with peculiar apically compressed crowns that are less expanded than the tooth roots, and ornamented with accessory basiapical keels [7], [33], [35]. Further, despite the damages possibly caused by the gastric corrosion, anatomical comparisons with other sphagesaurids corroborate the association of the cranial bones to the group [7], [33], [36]. The frontal and parietal are articulated and exposed in ventral aspect. The medial margins of both orbits and external supratemporal fenestrae are visible. Medial to the margin of each orbit, the frontal bears a slight longitudinal crest. The jugal has its medial surface exposed and the infraorbital, and postorbital infratemporal processes partially preserved; the latter of which is thin, as in other sphagesaurids [7], [36]. As in *Sphagesaurus montealtensis* [7] and *Caipirasuchus paulistanus* [36], the infraorbital process is slightly expanded dorsoventrally. This process bears a marked longitudinal ventral groove, forming the ectopterygoid articulation. Although no complete specimens of sphagesaurids are known, comparisons to other mesoeucrocodylians (*Caipirasuchus paulistanus* and *Araripesuchus gomesi*) suggest that the remains correspond to an animal of about 60 cm in length (see Text S1). Other isolated long bones of unknown affinity were found among the sphagesaurid cranial remains.

Despite the disarticulation of the tail and appendages, the complete gastralia indicates that the abdominal cavity of *Aplestosuchus sordidus* remained intact after burial [37]. Hence, the sphagesaurid remains positioned between the gastralia and the left ribs are best interpreted as abdominal contents or cololites. Cololites are the most direct evidence of prey selection in extinct animals [38], [39]; when preserved, these exceptional fossils provide reliable support for trophic relationships between the involved taxa. Excluding gastroliths from the definition of cololites, a single record of this kind is reported for fossil crocodyliforms, but it is only an unidentifiable material found in the abdominal region of a small crocodilian from the Green River Formation [40]. Accordingly, this is the first time that identifiable and unmistaken cololites have been described for the group.

Living crocodilians tend to eat smaller preys whole [41], increasing the possibility to dietary items be found as cololites, as we suggest. Likewise, extant crocodilians have the most acidic foregut measured for any vertebrate [42]. If this was also the case for older taxa, it could explain the scarcity of abdominal contents found in fossil crocodyliforms, as well as the loss of additional parts in the material reported here. Furthermore, the bone digestion efficiency in the group, as well as the corresponding size of the material, suggests that these fragments are very likely from the same individual, and consequently from the same taxon.

## Results and Discussion

### Affinity of Aplestosuchus sordidus

*Aplestosuchus sordidus* can be safely placed within Baurusuchidae based on several skull features exclusive of this clade [6], such as the quadrate depression, medial approximation of the prefrontals, rostral extension of palatines (not reaching the level of the rostral margin of suborbital fenestrae), cylindrical dorsal portion of the palatine bar, ridge on the ectopterygoid-jugal articulation, and supraoccipital with restricted thin transversal exposure in the caudalmost region of the skull roof.

**Figure 9:**
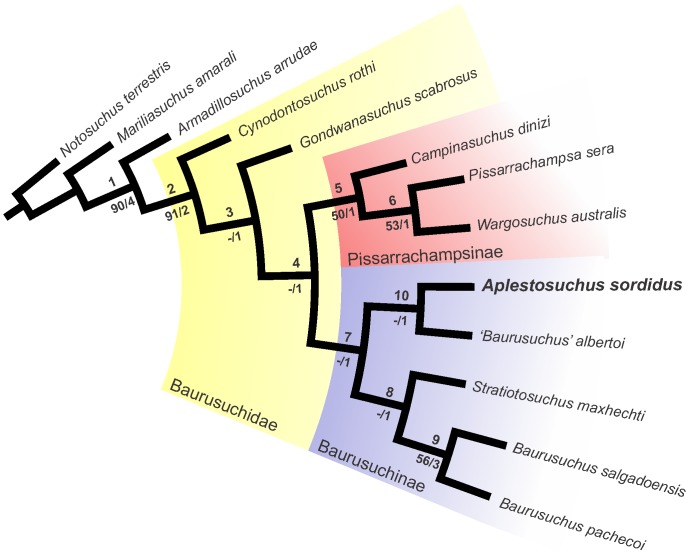
Single Most Parsimonious Tree depicting Baurusuchidae phylogenetic relationships and the position of *Aplestosuchus sordidus*. Bootstrap GC (50% cut) and decay values are shown below the clades, which are numbered as references for the synapomorphy list (Text S5).

A phylogenetic analysis was performed to investigate the affinities of *A. sordidus* to other baurusuchids (Figure 9). The single most parsimonious tree found is 119 steps long (CI = 0.72, RI = 0.73). Details of the phylogenetic analysis can be seen in the supporting information texts (Texts S2, S3, S4 and S5). The taxon-character matrix is an expanded version of that found in [6], which is focused on Baurusuchidae. Taxon sampling was expanded to incorporate *Aplestosuchus sordidus*, as well as *Campinasuchus dinizi* and *Gondwanasuchus scabrosus*. Eight new characters (67 to 74, see Text S2) were added to represent newly recognized morphological variations within Baurusuchidae.

This revaluation of baurusuchid phylogeny first aimed at defining the phylogenetic position of *Aplestosuchus sordidus*. However, the simultaneous incorporation of the recently described *Gondwanasuchus scabrosus* [24] and *Campinasuchus dinizi* [29] allows a global discussion of the interrelationships of the group. The general results of [6] are replicated here, with *Cynodontosuchus rothi* as the basal-most baurusuchid and “higher baurusuchids” (clade 4) forming a dichotomy composed of Pissarrachampsinae (clade 5) and Baurusuchinae (clade 7). *Aplestosuchus sordidus* is a baurusuchinae, forming with *Baurusuchus albertoi* (clade 10) the sister-clade to the group composed of *Stratiotosuchus maxhetchi* and *Baurusuchus pachecoi* + *B. salgadoensis*. This results in a paraphyletic arrangement for *Baurusuchus*. Indeed, if future studies confirm that position for *B. albertoi*, it should be removed from the genus *Baurusuchus*.

The basal placement of *Gondwanasuchus scabrosus* as the sister group of clade 4 suggests that ontogenetic biases may influence the study of baurusuchid phylogeny. The basal most baurusuchids, *Cynodontosuchus rothi* and *Gondwanasuchus scabrosus*, are also the smallest known members of the group and have the less sculptured skulls. In addition, *G. scabrosus* has paired nasals and *C. rothi* lacks the palatal median rogosity and its flanking row of foramina [6], both conditions that suggest osseous immaturity in comparison to other baurusuchid known species. Indeed, the lack of some traits that support the “higher baurusuchid” clade (clade 4) in these two taxa may also be related to their earlier ontogenetic stage. This is the case of their unique palatine bar (character 41, see Text S2), which is flat throughout in *C. rothi* and at its caudal portion in *G. scabrosus*. However, at this point, the distinctive suite of characters of each of these taxa (especially *G. scabrosus*) best supports their placement as unique taxonomic entities.

*Aplestosuchus sordidus* is easily distinguished from other baurusuchids by an exclusive suite of traits that validates its taxonomic uniqueness. Four characters found only in *A. sordidus* are briefly discussed hereafter. The midline crest on the nasal is restricted to the caudal portion of the fused bones, differently from the smooth surface seen in other baurusuchines. It also differs from the rugose broad depression seen on the dorsal surface of the nasal of pissarrachampsines and *Gondwanasuchus scabrosus*. Also, the rostral extension of the frontal midline crest in *A. sordidus* differs from the caudally restricted crest of other baurusuchines, and is also more rostrally developed than those of *P. sera* and *C. dinizi*. In the latter taxa, the crest does not reach the midline contact between prefrontals, whereas in *A. sordidus* it extends to the rostralmost portion of the frontal and reaches the prefrontal midline contact. The crested ventral surface of the palatine bar of *A. sordidus* is conspicuously different from the condition in other baurusuchids. As discussed above, the palatine bar of more has a flat ventral surface, resembling the usual condition for Crocodyliformes and Notosuchia. Moreover, the palatine bar of higher baurusuchids (node 4), has a restricted ventral surface and a cylindrical dorsal portion [6]. In *A. sordidus*, the lateral edges of the bar meet at the midline forming a sharp crest, and a ventral flat surface is virtually absent. Parachoanal fossae are not exclusive of baurusuchids [6], but these structures have distinctive morphologies within the clade, most notably in *P. sera.* In *A. sordidus* the parachoanal fossae are not divided in subfossae as in *P. sera* and are more rostrocaudally extended. Yet, the most distinct feature of the fossae in *A. sordidus* is its position. Whereas they intimately related to the parachoanal fenestrae in the other baurusuchid, close to the choanal septum, they are laterally displaced in *A. sordidus*, and located at the ventral surface of the pterygoid wing.

### Trophic relations of the Adamantina Formation fauna

Reaching up to four meters in length [43], baurusuchids were surely amongst the top predators of the South American Late Cretaceous ecosystems, surpassed only by large theropods [44]. Given its size relation to the prey, there is no good reason to consider the abdominal contents of LPRP/USP 0229a as more likely derived from scavenging, and the find corroborates the role of baurusuchids as predators, minimally capable of sizing prey of about one fourth of its size. Sphagesaurids (the prey item), are usually much smaller (but see [23]), and inferred to fill omnivorous niches [3], [7], [33]. These data suggest that sphagesaurids took the role of first to second level consumers in their communities, and took part in the diet of more apical predators, like baurusuchids (Figure S1).

Apart from Bolivia [45], and possibly in China [46], sphagesaurids are only known from the Adamantina Formation of Brazil, where six taxa were recovered. Whereas crocodilians are extraordinarily diverse, dinosaurs, except for the sauropods [47], are very rare in the Adamantina Formation. Except for isolated teeth and fragmentary tooth bearing bones, mostly attributed to abelisaurids and carcharodontosaurids [48], the record of theropods is restricted to isolated megaraptorid and unenlagiid vertebrae [49], [50], the first of which may come from an upper stratigraphic level. Further, ornithischian fossils are altogether absent from the Late Cretaceous of Brazil. This contrasts with coeval deposits in the Neuquén Basin in Argentina, which include several small bodied ornithischians and a great variety of theropods [51].

**Figure 10:**
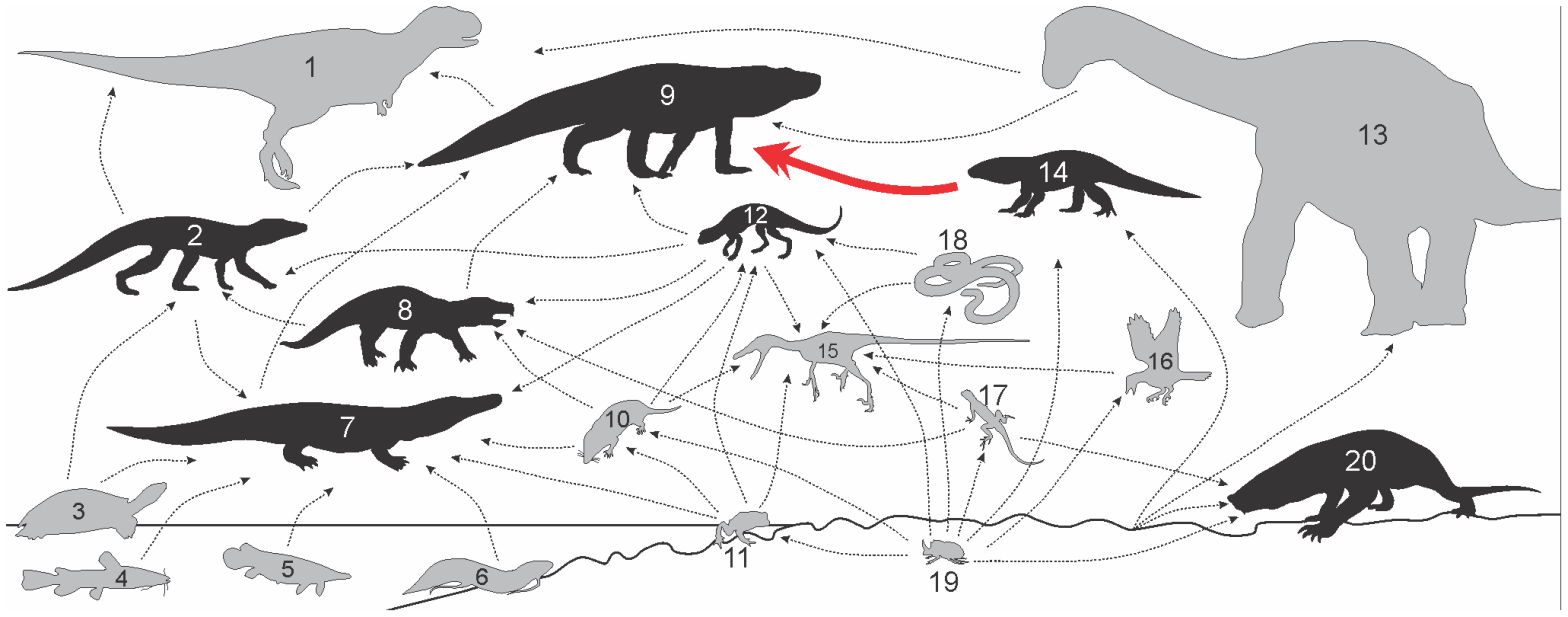
Inferred food web reconstruction of the Late Cretaceous fauna of Southeastern Brazil (Adamantina Formation). Crocodyliformes in black, and non-crocodyliforms in grey. Full red line depicts the reported baurusuchid-sphagesaurid interaction. Inferred interactions represented by dotted black lines. 1, Large theropods (Abelisauridae, Charcarodontosauridae, Megaraptora); 2, Peirosaurids (*Montealtosuchus arrudacamposi*, *Pepesuchus deiseae*); 3, Turtles (*Bauruemys elegans*, *Roxochelys wanderleyi*); 4-6, Fishes (Teleostei, Lepisosteidae, Dipnoi); 7, Trematochampsids (*Barreirosuchus franciscoi*); 8, “Notosuchians” (*Labidiosuchus amicum*, *Mariliasuchus amarali*, *M. robustus*, *Morrinhosuchus luziae*); 9, Baurusuchids (*Baurusuchus albertoi*, *B. pachecoi*, *B. salgadoensis*, *Campinasuchus dinizi*, *Gondwanasuchus scabrosus*, *Pissarrachampsa sera*, *Stratiotosuchus maxhetchi*); 10, Mammals; 11, Anurans; 12, *Adamantinasuchus navae*; 13, Sauropods (*Adamantisaurus mezzalirai*, *Aeolosaurus maximus*, *Gondwatitan faustoi*, *Maxakalisaurus topai*, Nemegtosauridae); 14, Other sphagesaurids (*Caipirasuchus paulistanus*, *Caryonosuchus pricei*, *Sphagesaurus huenei*, *S. montealtensis*); 15, Unenlagiines; 16, Birds (Enantiornithes); 17, Lizards (*Brasiliguana prudentis*); 18, Snakes (Anilioidea); 19, Insects (Coleoptera); 20, *Armadillosuchus arrudai*. (see Text S6 for complete references of Figure 10).

Although a better sampling of the Adamantina Formation is needed, the identified faunal composition stresses the atypical prevalence of Crocodyliformes, filling niches that are not usually occupied by the group, such as small to medium sized omnivores/herbivores and large terrestrial predators (Figure 10) [3], [52]. This unique ecological structure surely results from a series of factors, such as possible geographic isolation and the climate of this region, for which proposed models indicate subtropical arid to tropical conditions, hotter than in Patagonia (Neuquén Basin) [53]. Other Cretaceous ecosystems of Gondwana also demonstrate a great crocodyliform diversity (see [54]), indicating that some factors could be exclusive of Gondwanan landmasses. However, the richness of this group in the Adamantina Formation is exceptional and further investigation of factors is necessary for a better understanding of why and how the Mesoeucrocodylia flourished and became so diverse during the Late Cretaceous of Southeastern Brazil.

## Supporting Information

Figure S1 **Artistic reconstruction of** ***Aplestosuchus sordidus*** **preying on a sphagesaurid.** Drawing by Rodolfo Nogueira.
(TIF)

Text S1 **Size estimates for LPRP/USP 0229.**
(DOCX)

Text S2 **List of characters used in phylogenetic analysis.** Description of the 74 characters used in the phylogenetic analyses. The data matrix is in Text S4. Characters are new or adapted from previously published studies.
(DOCX)

Text S3 **List of taxa used in Parsimony Analysis.**
(DOCX)

Text S4 **Data Matrix.**
(DOCX)

Text S5 Synapomorphy list.
(DOCX)

Text S6 **References of the fossil record data for** **Figure 10** **.**
(DOCX)
