# Supplementary figures and images for: An Additional Baurusuchid from the Cretaceous of Brazil with Evidence of Interspecific Predation among Crocodyliformes
Source: PLoS One. 2014 May 8;9(5):e97138. doi: 10.1371/journal.pone.0097138 (PMC4014547; doi:10.1371/journal.pone.0097138)

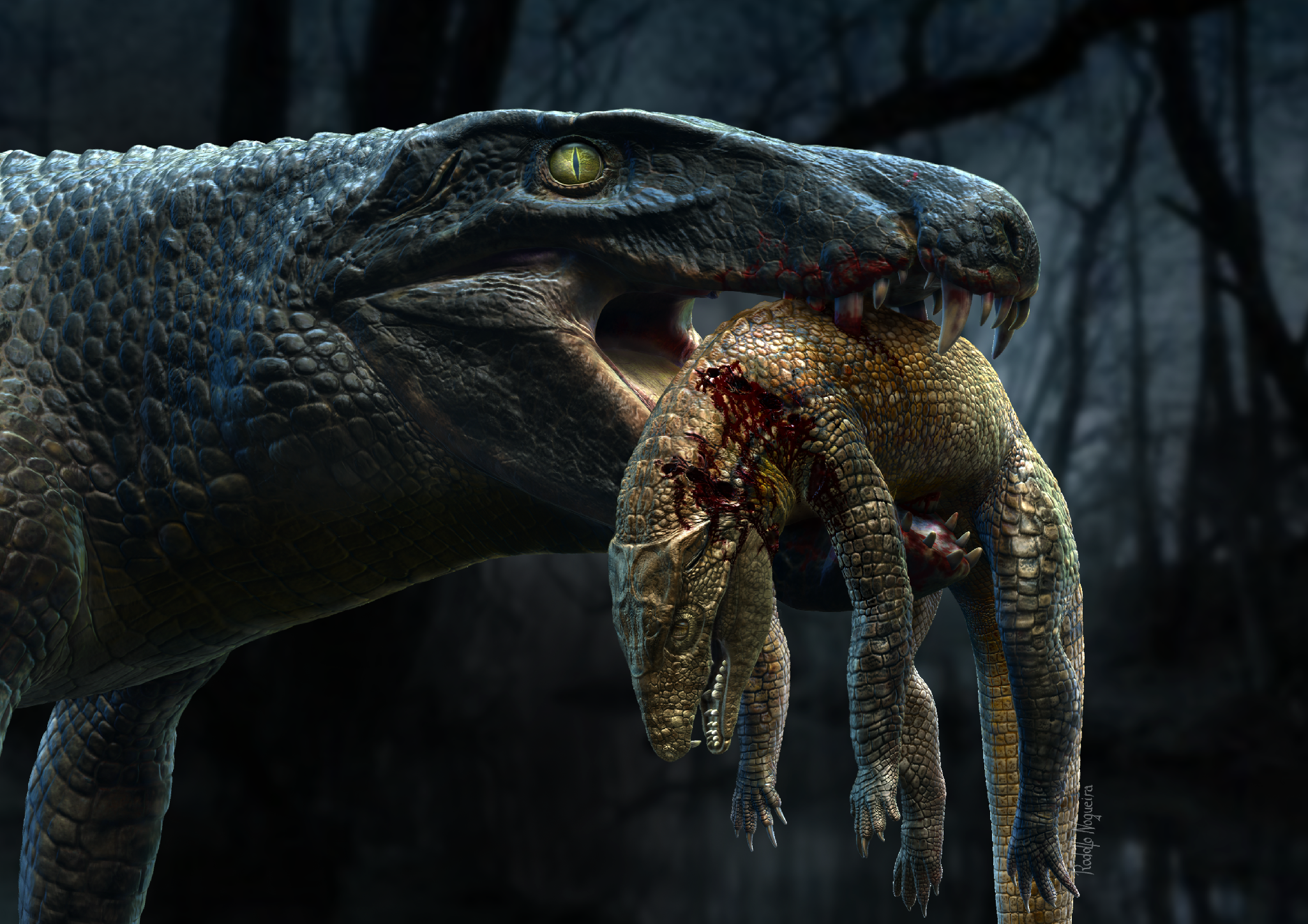

Supplement: Figure S1 — Artistic reconstruction of Aplestosuchus sordidus preying on a sphagesaurid. Drawing by Rodolfo Nogueira. (TIF) [file pone.0097138.s001.tif]
