# Supplementary material for: An Additional Baurusuchid from the Cretaceous of Brazil with Evidence of Interspecific Predation among Crocodyliformes
Source: PLoS One. 2014 May 8;9(5):e97138. doi: 10.1371/journal.pone.0097138 (PMC4014547; doi:10.1371/journal.pone.0097138)
Supplement: Text S1 — Size estimates for LPRP/USP 0229. (DOCX) [file pone.0097138.s002.docx]

**Text S1: Size estimates for LPRP/USP 0229**

LPRP/USP 0229a (*Aplestosuchus sordidus*):

Preserved length (tip of the skull to base of the tail) = 1,1 m

Body length estimate based on *Baurusuchus salgadoensis* [1] ≈ 2,3 m

LPRP/USP 0229b (Sphagesauridae indet.):

Parietal length = 25 mm

Skull length estimated based on *Cairpirasuchus paulistanus* [2] ≈ 95 mm

Body length estimated based on *Araripesuchus gomesi* [3] ≈ 55 cm

**References (Text S1)**

1. Vasconcellos FM, Carvalho IS (2010) Paleoichnological assemblage associated with *Baurusuchus salgadoensis* remains, a Baurusuchidae Mesoeucrocodylia from the Bauru Basin, Brazil (Late Cretaceous). Bulletin of the New Mexico Museum of Natural History and Science 51: 227–237.
2. Iori FV, Carvalho IS (2011) *Caipirasuchus paulistanus*, a new sphagesaurid (Crocodylomorpha, Mesoeucrocodylia) from the Adamantina Formation (Upper Cretaceous, Turonian–Santonian), Bauru Basin, Brazil. J Vertebr Paleontol 31: 1255-1264.
3. Maisey J (1991) Santana Fossils: An Illustrated Atlas. Neptune City: T.H.F. Publications. 459 p.
