# Supplementary material for: An Additional Baurusuchid from the Cretaceous of Brazil with Evidence of Interspecific Predation among Crocodyliformes
Source: PLoS One. 2014 May 8;9(5):e97138. doi: 10.1371/journal.pone.0097138 (PMC4014547; doi:10.1371/journal.pone.0097138)
Supplement: Text S2 — List of characters used in phylogenetic analysis. Description of the 74 characters used in the phylogenetic analyses. The data matrix is in Text S4. Characters are new or adapted from previously published studies. (DOCX) [file pone.0097138.s003.docx]

**Text S2: Character list**

Original sources are indicated after the characters and character state definitions. Characters 5, 11, 12, 22, 27, 32, and 41 are treated as additive and marked with a +.

1. Premaxilla-maxilla suture: in the lateral surface of rostrum (0), internalized in a notch for the reception of lower caniniform (1) [1].

2. Nasals: paired (0), partially or complete fused (1) [2].

3. Posterior portion of the nasal elevated above the dorsal surface of the maxillae, forming a sagittal bar: absent (0); present posteriorly (1) [3].

4. Dorsal surface of the nasal, posterior portion: rounded or flat (0), bearing a rugose broad depression (1) [3].

5. Prefrontal-prefrontal medial contact (+): absent (with a broad contact between nasal and frontal) (0) medial margin of prefrontals anteriorly convergent, almost touching each other (with a tiny contact between nasal and frontal) or touching each other anteriorly (1), contact present along mostly of the dorsal medial edge (2) [3].

6. Dorsal surfaces of prefrontal and anterior palpebral: continuous (0), central portion of the dorsal surfaces elevated, with a marked groove between them (1) [3].

7. Frontal: participates in the supratemporal fenestrae (0), excluded from the supratemporal fenestrae (1) [1].

8. Width of frontals between orbits relative to mid-length width across nasals: narrow (similar to width of nasals) (0); or broad (about twice the width of nasals) (1) [1].

9. Dorsal surface of frontal, posterior to orbits: flat or slightly concave (0), or markedly concave transversely (1) [4].

10. Midline longitudinal depression on anterior portion of frontal: absent (0), present (1) [3].

11. Frontal longitudinal ridge (+): absent (0), restricted to the posterior portion (1), extending anteriorly to the frontal mid-length (2) [1].

12. Support for the anterior palpebral bone (+): marked depression on prefrontal (0), marked depression on prefrontal and lacrimal forming an incipient lateral projection (1), marked depression on prefrontal and lacrimal forming a great lateral projection for the support of anterior palpebral (2) [5].

13. Supratemporal rims raised and hypertrophied: restricted to the median edge of the external supratemporal fenestra (0), extended to anterior edge of the external supratemporal fenestrae (1) [3].

14. Postorbital-quadratojugal contact in lateral view: restricted (0), broad contact between quadratojugal and the posterior portion of the postorbital descending flange (1) [1].

15. Lateral surface of the postorbital descending flange: flat (0), or concave (1) [3].

16. Postorbital-squamosal suture at the skull table in lateral view: straight line (0), squamosal anteriorly convex (1) [6].

17. Lateral margins of squamosal and postorbital in dorsal view: parallel (0), or diverging posteriorly (1) [7].

18. Squamosal prong horizontal extention: well developed (0), restricted (1) [1].

19. Squamosal lateral descending flange: obliquely directed (0), vertically directed with lateral edge medially concave and laterally convex (1), vertically directed and medially convex and laterally concave (2) [1].

20. Lacrimal duct external aperture: ventral to the corner formed by the dorsal and lateral lacrimal surfaces (0), at the corner (1) [3].

21. Contact between anterior and posterior palpebrals: only laterally, forming a supraorbital foramen (0), along the whole length without a foramen (1) [1].

22. Alveolar margin of maxilla in lateral view (+): almost straight (0), arched only anteriorly, below the enlarged caniniform tooth (1), arched along the entire alveolar margin (2) [1].

23. Maxillary palatal sagittal contact: smooth (0), bearing a longitudinal series of foramina (1) [3].

24. Dorsoventral depth of the jugal orbital portion in relation to infratemporal portion: almost the same depth (0), orbital portion twice the depth of the infratemporal portion (1) [1].

25. Dorsoventral depth of the jugal antorbital portion in relation to infraorbital portion: equal or lower (0), antorbital portion deeper than infraorbital portion (1) [8].

26. Posterior portion of the jugal orbital border: without a notch (0), with a marked ventral notch (1) [3].

27. Jugal outer surface (+): confluent along the entire length (0), infratemporal portion of jugal laterally displaced but not reaching the level of orbital anterior edge (1), infratemporal portion of jugal laterally displaced and reaching the level of orbital anterior edge (2) [8].

28. Jugal infratemporal bar: laterally flat (0), rod-shaped (1) [1].

29. Anterodorsal ramus of quadrate in ventral view: developed, forming more than 50% of the lateral edge of the internal supratemporal fenestra (0), restricted, forming less than 50% of the lateral edge of the internal supratemporal fenestra (1) [3].

30. Ventral border of quadratojugal and quadrate (lateral view): continuous (0), quadratojugal ventrally displaced, forming a notch between the bones (1) [3].

31. Anterior margin of distal portion of body of the quadrate: at a right angle to the anterolateral portion of the bone (0), slopes gently towards the anterolateral portion of the bone (1) [3].

32. Quadratojugal dorsal ramus in medial view ( +): ending ventrally, or at the same level, of the dorsal tip of laterotemporal fenestra (0), overcoming the dorsal tip of laterotemporal fenestra but not reaching the supratemporal fossa (1), overcoming the dorsal tip of laterotemporal fenestra and reaching the supratemporal fossa (2) [3].

33. Quadrate lateral depression: absent (0), present with the major axis dorsoventrally oriented (1), present with the major axis anteroposteriorly oriented and with quadratojugalquadrate suture within the depression (2) [3].

34. Quadrate fenestrae: visible in lateral view (0), internalized in otic notch (1) [3].

35. Lateral quadrate condyle: almost as anteroposteriorly wide as the medial condyle (0), or lateral quadrate condyle hemispherical (1) [3].

36. Muscle scar in the medial quadrate surface (ridge ‘A’): curved or almost straight (0), sigmoidal (1) [3].

37. Supraoccipital dorsal exposure: along the midline portion of posterior region of skull table (0), restricted to a thin surface attached to the posteriormost portion of parietal and squamosal (1) [3].

38. Posterior region of the auditory fossa: posteriorly open (0), bound posteriorly by a posteroventrolateral extension of the squamosal and exoccipital (1) [9].

39. Foramen incisivum: absent or small (0), enlarged (1) [3].

40. Anterior extension of palatine: passes the anterior margin of the suborbital fenestrae (0), does not reach the level of the anterior margin of suborbital fenestrae (1) [8].

41. Ventral face of palatine bar: flat and wide (0), flat and wide posteriorly and ventral surface restricted and dorsal portion cylindrical posteriorly (1) ventral surface restricted and dorsal portion cylindrical through (2) [3].

42. Medial palatal contact: smooth (0), rugose (1) [3].

43. Dorso-lateral surface of the ectopterygoid near jugal contact: smooth (0), bearing a series of irregularly-sized foramina (1) [3].

44. Row of foramina flanking the medial contact of the palatines: absent (0), present (1) [3].

45. Ridge on the ectopterygoid-jugal articulation: absent (0), present and continuous with the ventral ridge of the infratemporal portion of jugal (1), present but separated from the ventral ridge of the infratemporal portion of jugal by a notch at the posterior margin of the articulation (2) [3].

46. Ectopterygoid-jugal contact behind pterygoid wing in ventral view: rounded (0), medially angled (1) [3].

47. Ventral surface of the choanal septum: smooth (0), ridged (1) [3].

48. Major surface of the pterygoid wing: lateroventrally oriented (0), ventrally oriented (1) [3].

49. Posttemporal fenestrae: present (0), absent (1) [3].

50. Pterygoid parachoanal fenestra and depressions: absent (0), present (1) [10].

51. Basisphenoid ventral surface: continuous to surrounding bones (0), ventrally displaced and separated from the neighboring elements by a posteroventral step formed by a groove that separated the bone from the main occipital plane (forming a postchoanal pterygoidbasisphenoid tuberosity) (1) [3].

52. Anterior wall of the lateral Eustachian foramina: present, separating each foramen from the sulcus (0), absent, foramina open into the sulcus (1) [3].

53. Lateral Eustachian foramen: smaller than medial one (0), as large or larger than medial one (1) [3].

54. Paired ridges located medially on the ventral surface of the basisphenoid (originating at the anterior margins of lateral Eustachian foramina): absent (0), present and anteroventrally convergent (1), present and sub-parallel (2) [11].

55. Tooth mesial and distal carinae: smooth (0), bearing a pebbled surface (1), serrated (2) [12].

56. Premaxillary teeth: four (0), three (1) [13].

57. Enlarged premaxillary teeth: present (0), absent (1) [1].

58. Last premaxillary tooth hypertrophy: present (0), absent (1) [1].

59. Maxillary teeth: five or more (0), only four (1) [13].

60. Mandibular outer surface sculpture: present on dentary (0), present on dentary and splenial (1) [3].

61. Foramen intramandibularis oralis: small or absent (0), big and slot-like (1) [7].

62. Mandibular symphysis, orientation of anterior part: horizontal or slightly dorsally directed (0), forming an angle of approximately 45 degrees to the main axis of the jaw (1) [9].

63. Peg at the posterior surface of the mandibular symphysis formed by a ridged splenial suture: absent (0), present (1) [14].

64. Posteroventral symphyseal depressions: absent (0), present (1) [3].

65. M. pterygoideous posterior insertion: reaching the anterior external mandibular fenestra (0), in a marked depression on surangular-angular lateral surface on posterior edge of external mandibular fenestra (1) [1].

66. Ridged border of the angular medial face: overcomes the anterior edge of the mandibular fenestra (0), does not overcome the anterior edge of mandibular fenestra (1) [3].

67. (NEW) Dorsal skull profile: rostrum downturned with the inflexion point just in front of the orbit (0), profile relatively straight (1).

68. (NEW) Row of foramina dorsal to ectopterygoid-jugal suture: absent or small foramina (0), foramina well developed (1).

69. (NEW) Longitudinal sulcus in intertemporal bar: absent (0), present (1).

70. (NEW) Maxillae-palatines suture: round (0), transverse (1).

71. (NEW) Cylindrical portion of the palatine bar: same wideness through (0), constricted in the posterior portion (1).

72. (NEW) Maxillae-palatines transverse suture: without posterior process of maxillae (0) with a V-shaped posterior process of maxillae at midline (1).

73. (NEW) Series of foramina in angular below the mandibular fenestrae: absent (0), present (1).

74. (NEW) Dorsal margin of Mandibular fenestrae: round (0), straight line formed by surangular (1).

**References (Text S2)**

1. Clark JM (1994) Patterns of evolution in Mesozoic Crocodyliformes. In: Fraser NC, Sues H-D, editors. In the shadow of the dinosaurs New York: Cambridge University Press. pp. 84–97.
2. Gasparini Z, Pol D, Spalletti LA (2006) An unusual marine crocodyliform from the Jurassic-Cretaceous boundary of Patagonia. Science 311: 70–73.
3. Montefeltro FC, Larsson HCE, Langer MC (2011) A new baurusuchid (Crocodyliformes, Mesoeucrocodylia) from the Late Cretaceous of Brazil and the phylogeny of Baurusuchidae. PLoS ONE 6: e21916.
4. Riff D, Kellner AWA (2011) Baurusuchid crocodyliforms as theropod mimics: clues from the skull and appendicular morphology of *Stratiotosuchus maxhechti* (Upper Cretaceous of Brazil). Zool J Linn Soc 163: S37–S56.
5. Pol D, Gasparini Z (2009) Skull anatomy of *Dakosaurus andiniensis* (Thalattosuchia: Crocodylomorpha) and the phylogenetic position of Thalattosuchia. J Syst Palaeontol 7: 163–197.
6. Nascimento PM, Zaher H (2011) The skull of the Upper Cretaceous baurusuchid crocodile *Baurusuchus albertoi* Nascimento & Zaher 2010, and its phylogenetic affinities. Zool J Linn Soc 163: S116–S131.
7. Ortega F, Gasparini Z, Buscalioni AD, Calvo JO (2000) A new species of *Araripesuchus* (Crocodylomorpha, Mesoeucrocodylia) from the lower Cretaceous of Patagonia (Argentina). J Vertebr Paleontol 20: 57–76.
8. Pol D (1999) El Esqueleto Postcraneano de *Notosuchus terrestris* (Archosauria: Crocodyliformes) del Cretácico Superior de la Cuenca Neuquina y su Información Filogenética. Buenos Aires: Universidad de Buenos Aires. 158 p.
9. Sereno PC, Larsson HCE (2009) Cretaceous Crocodyliforms from the Sahara. Zookeys 28: 1–143.
10. Andrade MB, Bertini RJ (2008) A new *Sphagesaurus* (Mesoeucrocodylia: Notosuchia) from the Upper Cretaceous of Monte Alto City (Bauru Group, Brazil), and a revision of the Sphagesauridae. Hist Biol 20: 101–136.
11. Pol D, Norell MA (2004) A new crocodyliform from Zos Canyon, Mongolia. Am Mus Novit 3445: 1–36.
12. Ortega F, Buscalioni AD, Gasparini Z (1996) Reinterpretation and new denomination of *Atacisaurus crassiporatus* (Middle Eocene; Issel, France) as cf. *Iberosuchus* (Crocodylomorpha, Metasuchia). Geobios 29: 353–364.
13. Wu X-C, Sues H-D (1996) Anatomy and phylogenetic relationships of *Chimaerasuchus paradoxus*, an unusual crocodyliform reptile from the Lower Cretaceous of Hubei, China. J Vertebr Paleontol 16: 688–702.
14. Pol D, Apesteguia S (2005) New *Araripesuchus* remains from the Early Late Cretaceous (Cenomanian—Turonian) of Patagonia. Am Mus Novit 3490: 1–38.
