# Supplementary material for: An Additional Baurusuchid from the Cretaceous of Brazil with Evidence of Interspecific Predation among Crocodyliformes
Source: PLoS One. 2014 May 8;9(5):e97138. doi: 10.1371/journal.pone.0097138 (PMC4014547; doi:10.1371/journal.pone.0097138)
Supplement: Text S3 — List of taxa used in Parsimony Analysis. (DOCX) [file pone.0097138.s004.docx]

**Text S3: Taxa list**

*Institutional Abbreviations*

**CPP**, Centro de Pesquisas Paleontológicas Llewellyn Ivor Price, Uberaba, Brazil; **DGM**, Departamento Nacional de Produção Mineral, Rio de Janeiro, Brazil; **LPRP/USP**, Laboratório de Paleontologia de Ribeirão Preto-USP, Ribeirão Preto, Brazil; **MACN**, Museo Argentino de Ciências Naturales, Buenos Aires, Argentina **MLP**, Museo de La Plata, La Plata, Argentina; **MOZ**, Museo Professor J. Olsacher, Zapala, Argentina; **MPMA**, Museu de Paleontologia de Monte Alto, Monte Alto, Brazil; **MUCP**, Museo de Geología y Paleontología, Universidad Nacional del Comahue, Neuquén, Argentina; **MZSP**, Museu de Zoologia da USP, São Paulo, Brazil; **UFRJ**, Coleção de Paleontologia de Vertebrados da Universidade Federal do Rio de Janeiro no Rio de Janeiro, Rio de Janeiro, Brazil.

Taxa used in the phylogenetic analysis are listed below, followed by data sources. Holotypes and lectotypes marked with an asterisk.

**Outgroup**

*Armadillosuchus arrudae* → UFRJ DG 303R*, MPMA-64-0001-04.

*Mariliasuchus amarali* → UFRJ DG 50R*, UFRJ DG 105R, UFRJ DG 106R.

*Notosuchus terrestris* → MUCP 137, MUCP 147, MACN PV-RN-1037; MACN-PV-RN-1038; MACN-PV-RN-1045, MLP-64-IV-16-5*.

**Ingroup**

*Aplestosuchus sordidus* gen. et sp. nov. → LPRP/USP 0229*.

*Baurusuchus albertoi* → MZUSP-140*.

*Baurusuchus pachecoi* → [1, 2, 3, 4].

*Baurusuchus salgadoensis* → MPMA-62-0001-02*.

*Campinasuchus dinizi* → [5].

*Cynodontosuchus rothi* → MLP 64-IV-16-25*.

*Gondwanasuchus scabrosus* → [6].

*Pissarrachampsa sera* → LPRP/USP 0019*, LPRP/USP 0017, LPRP/USP 0018.

*Stratiotosuchus maxhechti* → DGM 1477-R*.

*Wargosuchus australis* → MOZ-PV 6134*.

**References (Text S3)**

1. Nascimento PM, Zaher HA (2010) A new species of *Baurusuchus* (Crocodyliformes, Mesoeucrocodylia) from the Upper Cretaceous of Brazil, with the first complete postcranial skeleton described for the family Baurusuchidae. Pap Avulsos Zool 50: 323–361.
2. Price LI (1945) A new reptil from the Cretaceous of Brazil. Notas Preliminares e Estudos – DGM 25: 1–8.
3. Nascimento PM, Zaher H (2011) The skull of the Upper Cretaceous baurusuchid crocodile *Baurusuchus albertoi* Nascimento & Zaher 2010, and its phylogenetic affinities. Zool J Linn Soc 163: S116–S131.
4. Pol D, Gasparini Z (2007) Crocodyliformes. In: Gasparini Z, Salgado L, Coria RA, editors. Patagonian Mesozoic Reptiles. Bloomington & Indianapolis: Indiana University Press. pp. 116–142.
5. Carvalho IS, Teixeira VPA, Ferraz MLF, Ribeiro LCB, Martinelli AG, et al. (2011) *Campinasuchus dinizi* gen. et sp. nov., a new Late Cretaceous baurusuchid (Crocodyliformes) from the Bauru Basin, Brazil. Zootaxa 2871: 19–42.
6. Marinho TS, Iori FV, Carvalho IS, Vasconcellos FM (2013) *Gondwanasuchus scabrosus* gen. et sp. nov., a new terrestrial predatory crocodyliform (Mesoeucrocodylia: Baurusuchidae) from the Late Cretaceous Bauru Basin of Brazil. Cretaceous Res 44: 104–111.
