# Supplementary material for: An Additional Baurusuchid from the Cretaceous of Brazil with Evidence of Interspecific Predation among Crocodyliformes
Source: PLoS One. 2014 May 8;9(5):e97138. doi: 10.1371/journal.pone.0097138 (PMC4014547; doi:10.1371/journal.pone.0097138)
Supplement: Text S4 — Data Matrix. (DOCX) [file pone.0097138.s005.docx]

**Text S4: Data Matrix**

Character states (0-2) are given for outgroup and ingroup taxa. Brackets enclose variable conditions; question marks indicate missing data; and dashes indicate inapplicable characters.

*Armadillosuchus arrudae*

01000000000100111000-????0111001001100????????????01101???????1???0?0?????

*Mariliasuchus amarali*

000000100001000[0/1]00001000-0100001001-0000000000010100001001000010000000—00

*Notosuchus terrestris*

00000?1000200000000-1000-000000?000?000000?00?0110000000000000000?0000—01

*Aplestosuchus sordidus*

11?01000102?0??011??12?11?21????[1/2][0/1]?011?121?12?10?11??1200100110111?1010011

*Baurusuchus albertoi*

?????????????111112?1??110211010111011????0?2??01???????????????10?1????11

*Baurusuchus pachecoi*

11???????????11101[1/2]??1111021?0101110?10121?11?100?11?220010?111?1010?11110

*Baurusuchus salgadoensis*

1110200110120111111012?1102110101110110121011??00?110120010111111111011100

*Campinasuchus dinizi*

11111?0?1?22111111[1/2]0?2?10021?11?101?110121?12?0111?1??20010111111?00110000

*Cynodontosuchus rothi*

1????????????????????[1/2]????????????????1100?0??????????211-0??111?????1-0??

Gondwanasuchus scabrosus

10111??0?1?2?1??????11?1002??01????????110?11?????????2001001111??00?10011

*Pissarrachampsa sera*

111110001122111[0/1]11[1/2]1121100211112200111?1211121011111122100111111??001100?0

*Stratiotosuchus maxhechti*

110020111012011011100211012110101100111121??201?1???0?2100011?11??101100??

*Wargosuchus australis*

11?111???1?2???????0??????????????????????????????????2?00????????????????
