# Supplementary material for: An Additional Baurusuchid from the Cretaceous of Brazil with Evidence of Interspecific Predation among Crocodyliformes
Source: PLoS One. 2014 May 8;9(5):e97138. doi: 10.1371/journal.pone.0097138 (PMC4014547; doi:10.1371/journal.pone.0097138)
Supplement: Text S5 — Synapomorphy list. (DOCX) [file pone.0097138.s006.docx]

**Text S5: Synapomorphy list**

Unambiguous synapomorphies are listed bellow for the nodes present in the single MPT (Figure 4).

**Node 1**

All trees:

Char. 6: 1 → 0

Char. 14: 0 → 1

Char. 16: 0 → 1

Char. 27: 0 → 1

Char. 28: 0 → 1

Char. 51: 0 → 1

**Node 2** Baurusuchidae

All trees:

Char. 0: 0 → 1

Char. 54: 1 → 2

**Node 3**

All trees:

Char. 40: 0 → 1

Char. 43: 0 → 1

**Node 4**

All trees:

Char. 21: 1 → 2

Char. 40: 1 → 2

Char. 41: 0 → 1

**Node 5** Pissarrachampsinae

All trees:

Char. 12: 0 → 1

Char. 29: 0 → 1

Char. 68: 0 → 1

**Node 6**

All trees:

Char. 57: 1 → 0

**Node 7** Baurusuchinae

All trees:

Char. 31: 1 → 0

Char. 33: 0 → 1

Char. 35: 1 → 0

Char. 46: 0 → 1

Char. 47: 1 → 0

**Node 8**

All trees:

Char. 4: 1 → 2

Char. 7: 0 → 1

**Node 9**

All trees:

Char. 44: 2 → 1

Char. 48: 1 → 0

Char. 70: 0 → 1

Char. 71: 0 → 1

**Node 10**

All trees:

Char. 67: 0 → 1
