# Supplementary material for: An Additional Baurusuchid from the Cretaceous of Brazil with Evidence of Interspecific Predation among Crocodyliformes
Source: PLoS One. 2014 May 8;9(5):e97138. doi: 10.1371/journal.pone.0097138 (PMC4014547; doi:10.1371/journal.pone.0097138)
Supplement: Text S6 — References of the fossil record data for Figure 10 . (DOCX) [file pone.0097138.s007.docx]

**Text S6: References of the fossil record data for Figure 10**

**1,** Large theropods (Abelisauridae, Charcarodontosauridae, Megaraptora) [1–3];

**2,** Peirosaurids (*Montealtosuchus arrudacamposi*, *Pepesuchus deiseae*) [4, 5];

**3,** Turtles (*Bauruemys elegans*, *Roxochelys wanderleyi*) [6–9];

**4–6,** Fishes (Teleostei, Lepisosteidae, Dipnoi) [10–13];

**7,** Trematochampsids (*Barreirosuchus franciscoi*) [14];

**8,** “Notosuchians” (*Labidiosuchus amicum*, *Mariliasuchus amarali*, *M. robustus*, *Morrinhosuchus luziae*) [15–18];

**9,** Baurusuchids (*Baurusuchus albertoi*, *B. pachecoi*, *B. salgadoensis*, *Campinasuchus dinizi*, *Gondwanasuchus scabrosus*, *Pissarrachampsa sera*, *Stratiotosuchus maxhetchi*) [19–25];

**10,** Mammals [10];

**11,** Anurans [26];

**12,** *Adamantinasuchus navae* [27];

**13,** Sauropods (*Adamantisaurus mezzalirai*, *Aeolosaurus maximus*, *Gondwatitan faustoi*, *Maxakalisaurus topai*, Nemegtosauridae) [28–32];

**14,** Other sphagesaurids (*Caipirasuchus paulistanus*, *Caryonosuchus pricei*, *Sphagesaurus huenei*, *S. montealtensis*) [33–36];

**15,** Unenlagiines [37];

**16,** Birds (Enantiornithes) [38, 39];

**17,** Lizards (*Brasiliguana prudentis*) [40, 41];

**18,** Snakes (Anilioidea) [42, 43];

**19,** Insects (Coleoptera) [44];

**20,** *Armadillosuchus arrudai* [45].

**References (Text S6)**

1. Azevedo RPF, Simbras FM, Furtado MR, Candeiro CRA, Bergqvist LP (2013) First brazilian carcharodontosaurid and other new theropod dinosaur fossils from the Campanian-Maastrichtian Presidente Prudente Formation, São Paulo State, southeastern Brazil. Cretaceous Res 40: 131–142.
2. Méndez AH, Novas FE, Iori FV (2012) First record of Megaraptora (Theropoda, Neovenatoridae) from Brazil. C R Palevol 11: 251–256.
3. Bittencourt JS, Langer MC (2011) Mesozoic dinosaurs from Brazil and their biogeographic implications. An Acad Bras Cienc 83: 23–60.
4. Campos DA, Oliveira GR, Figueiredo RG, Riff D, Azevedo SAK, et al. (2011) On a new peirosaurid crocodyliform from the Upper Cretaceous, Bauru Group, southeastern Brazil. An Acad Bras Cienc 83: 317–327.
5. Carvalho IS, Vasconcellos FM, Tavares SAS (2007) *Montealtosuchus arrudacamposi*, a new peirosaurid crocodile (Mesoeucrocodylia) from the Late Cretaceous Adamantina Formation of Brazil. Zootaxa 1607: 35–46.
6. França MAG, Langer MC (2006) Phylogenetic relationships of the Bauru Group turtles (Late Cretaceous of south-central Brazil). Revista Brasileira de Paleontologia 9: 1–9.
7. Kischlat E-E (1994) Observações sobre *Podocnemis elegans* Suáres (Chelonii, Pleurodira, Podocnemididae) do Neocretáceo do Brasil. Acta geologica leopoldensia 39: 345–351.
8. Price LI (1953) Os quelônios da Formação Bauru, Cretáceo terrestre do Brasil meridional. Boletim da Divisão de Geologia e Mineralogia DNPM 147: 1–34.
9. Suarez JM (1969) Um quelônio da formação Baurú. Anais XXIII Congresso Brasileiro de Geologia. Salvador. pp. 167–176.
10. Bertini RJ, Marshall LG, Gayet M, Brito P (1993) Vertebrate faunas from the Adamantina and Marilia formations (Upper Bauru Group, Late Cretaceous, Brazil). Neues Jahrb Geol Palaontol Abh 188: 71–101.
11. Gayet M, Brito PM (1989) Ichtyofaune Nouvelle du Crétacé Supérieur du Groupe Bauru (états de São Paulo et Minas Gerais, Brésil). Geobios 22: 841–847.
12. Martinelli AG, Ribeiro LCB, Marinho TS, Vasconcellos FM, Neto FM, et al. (2012) Sobre a presença de *Atractosteus* (Lepisosteiformes) na Formação Adamantina, Cretáceo Superior de Campina Verde, MG. Boletim de Resumos da Reunião Regional da Sociedade Brasileira de Paleontologia – Paleo 2012. Ribeirão Preto. pp. 23.
13. Nava WR, Brito PM (2008) Restos de peixes da Formação Adamantina, Cretáceo Superior da Bacia Bauru, regiões de Marília e Presidente Prudente, Estado de São Paulo. VI Simpósio Brasileiro de Paleontologia de Vertebrados. Ribeirão Preto. pp. 144.
14. Iori FV, Garcia KL (2012) Barreirosuchus franciscoi, um novo Crocodylomorpha Trematochampsidae da Bacia Bauru, Brasil. Revista Brasileira de Geociências 42: 397–410.
15. Carvalho IS, Bertini RJ (199) *Mariliasuchus*: um novo Crocodylomorpha (Notosuchia) do Cretáceo da Bacia Bauru. Geología Colombiana 24: 83–105.
16. Iori FV, Carvalho IS (2009) *Morrinhosuchus luziae*, um novo Crocodylomorpha Notosuchia da Bacia Bauru, Brasil. Revista Brasileira de Geociências 39: 717–725.
17. Kellner AWA, Figueiredo RG, Azevedo SAK, Campos DA (2011) A new cretaceous notosuchian (Mesoeucrocodylia) with bizarre dentition from Brazil. Zool J Linn Soc 163: S109–S115.
18. Nobre PH, Carvalho IS, Vasconcellos FM & Nava WR (2007) *Mariliasuchus robustus*, um novo Crocodylomorpha (Mesoeucrocodylia) da Bacia Bauru, Brasil. Anuário do Instituto de Geociências 30: 38–49.
19. Montefeltro FC, Larsson HCE, Langer MC (2011) A new baurusuchid (Crocodyliformes, Mesoeucrocodylia) from the Late Cretaceous of Brazil and the phylogeny of Baurusuchidae. PLoS ONE 6: e21916.
20. Nascimento PM, Zaher HA (2010) A new species of *Baurusuchus* (Crocodyliformes, Mesoeucrocodylia) from the Upper Cretaceous of Brazil, with the first complete postcranial skeleton described for the family Baurusuchidae. Pap Avulsos Zool 50: 323–361.
21. Marinho TS, Iori FV, Carvalho IS, Vasconcellos FM (2013) *Gondwanasuchus scabrosus* gen. et sp. nov., a new terrestrial predatory crocodyliform (Mesoeucrocodylia: Baurusuchidae) from the Late Cretaceous Bauru Basin of Brazil. Cretaceous Res 44: 104–111.
22. Campos DA, Suarez JM, Riff D, Kellner AWA (2001) Short note on a new Baurusuchidae (Crocodyliformes, Metasuchia) from the Upper Cretaceous of Brazil. Boletim do Museu Nacional 57: 1–7.
23. Carvalho IS, Campos ACA, Nobre PH (2005) *Baurusuchus salgadoensis*, a new Crocodylomorpha from the Bauru Basin (Cretaceous), Brazil. Gondwana Res 8: 11–30.
24. Carvalho IS, Teixeira VPA, Ferraz MLF, Ribeiro LCB, Martinelli AG, et al. (2011) *Campinasuchus dinizi* gen. et sp. nov., a new Late Cretaceous baurusuchid (Crocodyliformes) from the Bauru Basin, Brazil. Zootaxa 2871: 19–42.
25. Price LI (1945) A new reptil from the Cretaceous of Brazil. Notas Preliminares e Estudos – DGM 25: 1–8.
26. Carvalho AB, Zaher H, Nava WR (2004) A new anuran (Lissamphibia: Tetrapoda) from the continental Upper Cretaceous Bauru Basin, State of São Paulo, Brazil. Abstracts of Papers Sixty-Fourth Annual Meeting Society of Vertebrates Paleontology. Denver. pp. 45A.
27. Nobre PH, Carvalho IS (2010) *Adamantinasuchus navae*: A new Gondwanan Crocodylomorpha (Mesoeucrocodylia) from the Late Cretaceous of Brazil. J S Am Earth Sci 29: 346–353.
28. Santucci RM, Arruda-Campos AC (2011) A new sauropod (Macronaria, Titanosauria) from the Adamantina Formation, Bauru Group, Upper Cretaceous of Brazil and the phylogenetic relationships of Aeolosaurini. Zootaxa 3085: 1–33.
29. Avilla LS, Candeiro CRA, Nava WR (2005) Unusual sauropod dentary from the Adamantina Formation (Turonian-Santonian) of São Paulo, Brazil, with some comments on its relationships and paleobiogeography. Boletim de Resumos II Congresso Latino-Americano de Paleontologia de Vertebrados. Rio de Janeiro. pp. 42–43.
30. Kellner AWA, Azevedo SAK (1999) A new sauropod dinosaur (Titanosauria) from the Late Cretaceous of Brazil. Proceedings of the Second Gondwana Dinosaur Symposium 15: 111–142.
31. Kellner AWA, Campos DA, Azevedo SAK, Trotta MNF, Henriques DDR, et al. (2006) On a new titanosaur sauropod from the Bauru Group, Late Cretaceous of Brazil. Boletim do Museu Nacional. Nova Série Geologia 74: 1–31.
32. Santucci RM, Bertini RJ (2006) A new titanosaur from western São Paulo State, Upper Cretaceous Bauru Group, South-East Brazil. Palaeontology 49: 59–66.
33. Andrade MB, Bertini RJ (2008) A new *Sphagesaurus* (Mesoeucrocodylia: Notosuchia) from the Upper Cretaceous of Monte Alto City (Bauru Group, Brazil), and a revision of the Sphagesauridae. Hist Biol 20: 101–136.
34. Kellner AWA, Campos DA, Riff D, Andrade MB (2011) A new crocodylomorph (Sphagesauridae, Notosuchia) with horn-like tubercles from Brazil. Zool J Linn Soc 163: S57–S65 (2011).
35. Iori FV, Carvalho IS (2011) *Caipirasuchus paulistanus*, a new sphagesaurid (Crocodylomorpha, Mesoeucrocodylia) from the Adamantina Formation (Upper Cretaceous, Turonian–Santonian), Bauru Basin, Brazil. J Vertebr Paleontol 31: 1255–1264.
36. Price LI (1950) On a new crocodilian, *Sphagesaurus*, from the Cretaceous of the State of São Paulo, Brazil. An Acad Bras Cienc 22: 77–85.
37. Candeiro CR, Cau A, Fanti F, Nava W, Novas FE (2012) First evidence of an unenlagiid (Dinosauria, Theropoda, Maniraptora) from the Bauru Group, Brazil. Cretaceous Res 37: 223–226.
38. Alvarenga H, Nava WR (2005) Aves Enantiornithes do Cretáceo Superior da Formação Adamantina do Estado de São Paulo. Boletim de Resumos II Congresso Latino-Americano de Paleontologia de Vertebrados. Rio de Janeiro. pp. 20.
39. Azevedo RP, Vasconcellos PL, Candeiro CRA, Bergqvist LP (2007) Restos microscópicos de vertebrados fósseis do Grupo Bauru (Neocretáceo), no oeste do Estado de São Paulo. In: Carvalho IS, Cassab RCT, Schwanke CP, Carvalho MA, Fernandes AC, et al. editors. Paleontologia: Cenários de Vida. 1ed. Rio de Janeiro: Interciência Ltda. pp. 541-549.
40. Candeiro CRA, Nava WR, Martinelli AG, Forasiepi A, Scanferla CA, et al. (2009) New lizard record (Diapsida, Lepidosauria) from the Upper Cretaceous Adamantina Formation, Brazil. Bulletin of Geosciences (Praha) 84: 573–576.
41. Nava WR, Martinelli AG (2011) A new squamate lizard from the Upper Cretaceous Adamantina Formation (Bauru Group), São Paulo State, Brazil. An Acad Bras Cienc 83: 291-299.
42. Fachini TS, Hsiou AS (2011) Presence of an “Anilioid” Snake from the Late Cretaceous of Adamantina Formation, Brazil. Ameghiniana, Resúmenes IV Congreso Latinoamericano de Paleontología de Vertebrados. San Juan. pp. R164.
43. Zaher H, Langer MC, Fara E, Carvalho IS, Arruda JT (2003) A mais antiga serpente (Anilioidea) brasileira: Cretáceo superior do Grupo Bauru, General Salgado, Brasil. Paleontologia em Destaque (Paleo 2003) 44: 52.
44. Carvalho IS, Gracioso DE, Fernandes ACS (2009) Uma câmara de coleóptero (*Coprinisphaera*) do Cretáceo Superior, Bacia Bauru. Revista Brasileira de Geociências 39, 679–684.
45. Marinho TS, Carvalho IS (2009) An armadillo-like sphagesaurid crocodyliform from the Late Cretaceous of Brazil. J S Am Earth Sci 27: 36–41.
